# Small Extracellular Vesicles from adipose derived stromal cells significantly attenuate in vitro the NF-κB dependent inflammatory/catabolic environment of osteoarthritis

**DOI:** 10.1038/s41598-020-80032-7

**Published:** 2021-01-13

**Authors:** Carola Cavallo, Giulia Merli, Rosa Maria Borzì, Nicoletta Zini, Stefania D’Adamo, Michele Guescini, Brunella Grigolo, Alessandro Di Martino, Spartaco Santi, Giuseppe Filardo

**Affiliations:** 1 Laboratorio RAMSES, IRCCS Istituto Ortopedico Rizzoli, Via di Barbiano 1/10, 40136 Bologna, Italy; 2 Applied and Translational Research Center (ATRc), IRCCS Istituto Ortopedico Rizzoli, Via di Barbiano 1/10, 40136 Bologna, Italy; 3 Laboratorio di Immunoreumatologia e Rigenerazione Tissutale, IRCCS Istituto Ortopedico Rizzoli, Via di Barbiano 1/10, 40136 Bologna, Italy; 4 CNR Institute of Molecular Genetics “Luigi Luca Cavalli-Sforza”, Unit of Bologna, Via di Barbiano 1/10, 40136 Bologna, Italy; 5 IRCCS Istituto Ortopedico Rizzoli, Via di Barbiano 1/10, 40136 Bologna, Italy; 6 Department of Medical and Surgical Sciences, University of Bologna, Bologna, Italy; 7 Department of Biomolecular Sciences, University of Urbino Carlo Bo, Urbino, Italy; 8 Clinica Ortopedica e Traumatologica 2, IRCCS Istituto Ortopedico Rizzoli, Bologna, Italy

**Keywords:** Molecular biology, Stem cells, Rheumatology

## Abstract

The therapeutic ability of Mesenchymal Stem/Stromal Cells to address osteoarthritis (OA) is mainly related to the secretion of biologically active factors, which can be found within their secreted Extracellular Vesicles including small Extracellular Vesicles (sEV). Aim of this study was to investigate the effects of sEV from adipose derived stromal cells (ADSC) on both chondrocytes and synoviocytes, in order to gain insights into the mechanisms modulating the inflammatory/catabolic OA environment. sEV, obtained by a combined precipitation and size exclusion chromatography method, were quantified and characterized, and administered to chondrocytes and synoviocytes stimulated with IL-1β. Cellular uptake of sEV was evaluated from 1 to 12 h. Gene expression and protein release of cytokines/chemokines, catabolic and inflammatory molecules were analyzed at 4 and 15 h, when p65 nuclear translocation was investigated to study NF-κB pathway. This study underlined the potential of ADSC derived sEV to affect gene expression and protein release of both chondrocytes and synoviocytes, counteracting IL-1β induced inflammatory effects, and provided insights into their mechanisms of action. sEV uptake was faster in synoviocytes, where it also elicited stronger effects, especially in terms of cytokine and chemokine modulation. The inflammatory/catabolic environment mediated by NF-κB pathway was significantly attenuated by sEV, which hold promise as new therapeutic strategy to address OA.

## Introduction

Osteoarthritis (OA) is one of the most common and disabling conditions, with worldwide estimates showing that 9.6% of men and 18.0% of women over 60 years old suffer for symptomatic OA^1^. Progressive degradation of articular cartilage and subchondral bone leads to loss of joint function and pain, significantly impairing patient quality of life^2,3^. Moreover, OA chronicity leads to walking disability and increases vascular diseases causing, in the end, a 1.55 higher risk ratio of death compared to the general population^4^. OA prevalence is further increasing due to the aging population while its social impact is more dramatic than in the past due to the promotion of an active life-style also in the older population^5^. Thus, OA is a common cause of medical consultation^6^, presenting several challenges to the physician. In fact, from one side end-stage OA is frequently addressed with prosthetic metal resurfacing and invasive procedures offering suboptimal results and potentially entailing serious risks and complications^7^, and on the other hand more than half of all persons with symptomatic knee OA are younger than 65 years of age. Arthroplasty is less indicated for younger patients, where conservative measures are therefore advisable^8^.

As many of these younger people affected by OA will live for 3 decades or more, there is substantially more time for greater disability to occur, which emphasizes the need for the development of innovative treatment strategies^8^. To address OA mitigating the clinical, economic, and social burden of this disease, new biological injective treatment options have been recently proposed^9,10^. Among these, cell-based procedures hold promise, with Mesenchymal Stem/Stromal Cells (MSCs) being increasingly studied as a minimally invasive approach to modulate the articular inflammation while stimulating the regeneration processes of the joint tissues, in the end re-establishing joint homeostasis^11,12^. While definite trends can be observed with regard to the use in the current clinical practice, where cells are mostly being administered by i.a. injection and cell concentrates and especially those derived from the adipose tissue are increasingly used^12^, another strategy is gaining interest to exploit the potential of MSCs while avoiding some of the regulatory and practical limitations related to the cell injection approach. In fact, it has been demonstrated that the therapeutic ability of MSCs is mainly related to their secretion of biologically active factors^13^, and both small Extracellular Vesicles (sEV) and exosomes (EXO), a subset of cell-secreted vesicles sharing a 50–150 nm size, are a key part of the MSC secretome^14,15^, with EXO even being able to transfer cargo from donor to acceptor cells thus modifying their behavior^16^. However, while preliminary studies underlined the potential of this field, the mechanisms and effects of sEV and EXO in the OA environment remain to be elucidated.

Thus, the aim of this study was to investigate the effects of extracellular vesicles from adipose derived stromal cells (ADSC) on both chondrocytes and synoviocytes, in order to gain insights into the mechanisms modulating the inflammatory/catabolic OA environment. Moreover, since the boundary between sEV and EXO of similar size is not yet very clear, requiring a thorough analysis of the involvement of the Multivesicular bodies pathway^15^, we here focused on sEV.

## Materials and methods

### Adipose tissue-derived stromal cells

ADSC were obtained from microfractured adipose tissue collected from disposal material during regenerative medicine treatment of the knee of three donors (two men and one woman, aged 58 ± 11 years).

The study was conducted in accordance with the 1975 Declaration of Helsinki. Samples were obtained after informed consent from all patients, according to the protocol detailed in the study “ADIPO_CELL” (Prot.gen.n.ro 0009545, 3 October 2017) approved by the Ethics Committee of Istituto Ortopedico Rizzoli.

For each primary culture, details about patient sex, age, and BMI were recorded. Briefly, adipose tissue samples were washed with phosphate-buffered saline (PBS), isolated by enzymatic digestion with 0.05% type I collagenase (SIGMA-ALDRICH, St. Louis, MO) at 37 °C for 1 h and filtered through a 100 μm cell strainer (BD BIOSCIENCES, Bedford, MA, USA). Then, cells of the Stromal Vascular Fraction were washed with α-MEM (SIGMA-ALDRICH) containing 15% FBS, seeded into culture flasks (20 × 10^3^ cells/cm^2^) and incubated at 37 °C in a 5% CO_2_ atmosphere in α-MEM with 15% FBS. At confluence, ADSC were detached by treatment with trypsin–EDTA. Then the cells were seeded at 2 × 10^3^ cells/cm^2^ in α-MEM containing 15% of exosome-depleted FBS (GIBCO, LIFE TECHNOLOGIES, Grand Island, NY, USA) and cultured at 37 °C in 5% CO_2_ up to passage 4. Conditioned medium (CM) of ADSC cultures was collected from passage 2 to passage 4 every third day, pooled, centrifuged and stored in sterile conditions at − 80 °C until use.

An EXO-depleted FBS (GIBCO Fetal Bovine Serum, exosome-depleted Cat #LSA2720801 (90% depleted, according to available technical information) was used to culture ADSC, so that the sEV collected and used in the following experiments could closely reflect the trophic activity of ADSC.

### sEV isolation

Purified sEV were obtained from the CM of three different ADSC cultures using a protocol that combines precipitation/centrifugation and size exclusion chromatography following the manufacturer’s instructions (Exo-spin, CELL GUIDANCE SYSTEMS) to improve recovery and specificity^17^. Briefly, CMs were initially spun at 300×*g* for 10 min at 4 °C and then the obtained supernatant at 16,000×*g* for 30 min at 4 °C to remove cells and cellular debris. Successively, supernatants were incubated with Exo-spin Buffer (CELL GUIDANCE SYSTEMS) overnight at 4 °C. After a centrifugation at 16,000×*g* for 1 h at 4 °C, the sEV-containing pellet was resuspended in PBS, applied to the top of an Exo-spin column and centrifuged at 50 × *g* for 60 s. To elute the sEV, a further centrifugation at 50×*g* for 60 s with 200 µl of PBS was used. sEV were stored at − 80 °C until their use.

### sEV quantification and surface epitope characterization

sEV isolated from the CM were quantified using a NanoOrange Protein Quantification Kit (LIFE TECHNOLOGIES). Briefly, sEV were diluted 1:100 in the 1× NanoOrange working solution and incubated at 90 °C for 10 min. Then, the samples were cooled for at least 20 min and the fluorescence was read at 470ex–570em nm wavelength, using a Spectra Max Gemini plate fluorometer (MOLECULAR DEVICES, Sunnyvale, CA). To characterize sEV surface epitopes, 5 μg of sEV were analyzed using the MACSPlex Exosome Kit (MILTENYI BIOTEC) that allows the detection of 37 surface markers and two isotype controls. sEV were incubated with MACSPlex Exosome Capture Beads and with MACSPlex Exosome Detection Reagent CD9, CD63, and CD81 for 1 h at RT. Then, 1 ml of MACSPlex Buffer was added to each sEV containing tube, and left for 15 min at RT. The sEV bound to the Capture Beads were washed by centrifuging at 3000×*g* for 5 min, and then resuspended in 150μL of MACSPlex Buffer. Samples were analyzed with a FACS Canto II (BD BIOSCIENCES). Surface markers were calculated subtracting the median signal intensity of each bead of the control sample from the signal intensities of the respective beads incubated with sEV.

### sEV characterization by nanotracker analysis, transmission electron microscopy and western blot

Additional analyses were as described below, and carried out on essentially the same samples to provide correlated informations. sEV size distribution curves and concentration measurements were undertaken by Nanoparticle Tracking Analysis. sEV were diluted to approximately 1 mL of PBS, loaded into the sample chamber of an LM10 unit (NANOSIGHT, Malvern, UK) and three videos of either 30 or 60 s were recorded of each sample. Analysis was performed with NTA 3.1 software (NANOSIGHT) and data were presented as the mean ± SD of the three video recordings. When samples contained high numbers of particles, they were diluted before analysis and the relative concentration was then calculated according to the dilution factor. Control 100 and 400 nm beads were supplied by MALVERN INSTRUMENTS Ltd. (Malvern, UK)^18^.

For transmission electron microscopy analysis, sEV preparations were mixed with an equal volume of 4% paraformaldehyde (PFA) (SIGMA-ALDRICH) in phosphate buffer. 5 µl of suspension was deposited on 200 mesh Formvar-carbon-coated grids and left to absorb for 20 min at room temperature. Then the samples were fixed in 1% glutaraldehyde (SIGMA-ALDRICH) in phosphate buffer, contrasted with uranyl oxalate, pH 7.0, and embedded in a mixture of 4% uranyl acetate and 2% methyl cellulose (25 cps; SIGMA-ALDRICH)^19^.

The grids were examined with a ZEISS EM109 transmission electron microscope (ZEISS, Oberkochen, Germany). Images were captured using a NIKON digital camera Dmx 1200F (NIKON Corporation, Tokyo, Japan) and ACT-1 software (NIKON Corporation). More than 100 sEV were measured to provide information on their size distribution.

Western blot analysis, was carried out essentially as detailed in^20^ to rule out the presence of cellular organelle contamination (ER/Golgi) as recommended in^17^. sEV samples (20 µg) were run along 1.5 × 10^5^ ADSC as a positive control. sEV and ADSC were extracted with 20 µl of radioimmunoprecipitation (RIPA) buffer with the addition of benzonase and protease inhibitor cocktail (PIC; SIGMA-ALDRICH). The composition of the buffer was as follows: 50 mM Tris–HCl, pH 7.4, 150 mM NaCl, 1% Nonidet P-40, 0.1%SDS, 0.5% Na deoxycholate, 1 mM NaF, 1 mM Na_3_VO_4_, 1 mM PMSF, 1:200 PIC, and 100U/mL benzonase. Total cellular and sEV lysates were obtained by solubilizing samples with RIPA buffer followed by vortexing. Then, the samples were loaded in the wells of a Nu-Page precast 4%–10% polyacrylamide gel (INVITROGEN), along with a proper molecular weight marker (Novex Sharp Pre-stained Protein Standard, INVITROGEN), run and subsequently transferred onto polyvinylidene fluoride membranes by a dry electroblotting method using I-Blot (INVITROGEN). Then, the blots were subjected to immunodetection exploiting the SNAP-ID 2.0 device (MERCK MILLIPORE). The following primary antibodies were used: calnexin (mouse monoclonal, clone AF18, INVITROGEN, used at 1 µg/ml), Golgin 97 (mouse monoclonal, clone CDF4, INVITROGEN, used at 0.2 µg/ml). Signals were detected with appropriate HRP conjugated secondary antibodies (JACKSON IMMUNORESEARCH) and revealed with ECL Select (AMERSHAM), using the CCD camera acquisition system of a ChemiDOC apparatus (BIO-RAD LABORATORIES).

### Chondrocyte and synoviocyte isolation

Chondrocytes (1 man and 2 women, aged 66 ± 8.7 years) and synoviocytes (2 men, aged 69 ± 1 years) were isolated from the knees of patients undergoing joint replacement surgery.

The study was conducted in accordance with the 1975 Declaration of Helsinki. Samples were obtained after informed consent from all patients, according to the protocol detailed in the study “ADIPO_CELL” (Prot.gen.n.ro 0009545, 3 October 2017) approved by the Ethics Committee of Istituto Ortopedico Rizzoli.

Briefly, cartilage and synovial tissues were washed twice with PBS and minced into small pieces. Chondrocytes were obtained from three patients and isolated by sequential enzymatic digestions: 1 h with pronase (Sigma) and 1–2 h with 0.2% collagenase (SIGMA-ALDRICH) at 37 °C. Then, isolated chondrocytes were filtered by 100 µm and 70 µm nylon meshes, washed, and centrifuged. Cells were seeded at 8 × 10^3^ cells/cm^2^ in T150 flasks and cultured under conventional monolayer culture conditions in DMEM (SIGMA-ALDRICH) with 10% FCS. Chondrocytes from each patient were frozen at passage 0, collected upon confluency. Synoviocytes were isolated from the synovium of two patients. The tissue was minced into small pieces and cultured in DMEM (SIGMA-ALDRICH) with 10% FCS for two weeks in order to allow the release of the cells. Synoviocytes from each patient were frozen at passage 0. When the planned number of samples were collected, stored chondrocytes and synoviocytes were thawed and seeded at 8 × 10^3^ cells/cm^2^ in T150 flasks. Once enough cells (either chondrocytes or synoviocytes) were available, they were pooled and used for the experiments as described below. In the experiments carried out to assess sEV ability to attenuate the effects of IL-1β treatment of chondrocytes and synoviocytes a non EXO depleted FBS was used. Indeed, IL-1β delivery to these cells as a tool to reproduce OA pathophysiology is widely accepted as a powerful way to induce strong inflammatory and catabolic effects independently of the EXO amount contained in the FBS. Since the latter is shared by both IL-1β treated and IL-1β + sEV treated cells we believe that the functional effects of sEV addition could be clearly and unambiguously assessed.

### Monoculture and co-culture experiments

For monoculture experiments, chondrocytes and synoviocytes were thawed and seeded in 24-well plates at high density (2 × 10^5^/cm^2^ and of 6 × 10^4^/cm^2^, respectively) for 3 days in DMEM (SIGMA-ALDRICH) with 10% FCS. Successively, cells were treated with IL-1β (5 ng/mL, R&D SYSTEMS, Minneapolis, MN) for 18 h to mimic an inflammatory environment. Then the IL-1β containing medium was substituted with fresh 10% FBS—DMEM containing 10 μg/mL of sEV (sEV group) or without sEV (CTR group). In detail, the effects of three different purified sEV preparations were evaluated on pooled chondrocytes and pooled synoviocytes in either monoculture or co-culture. Co-cultures were set up by seeding 2 × 10^5^/cm^2^ chondrocytes in the lower chamber of a 24 well plate and 6 × 10^4^/cm^2^ synoviocytes in the transwell (0.4 μm pore size, CORNING, Toledo, OH) for 3 days in DMEM (SIGMA-ALDRICH) with 10% FCS. Successively, cells were treated with IL-1β (5 ng/mL, R&D SYSTEMS, Minneapolis, MN) for 18 h to mimic an inflammatory environment. The following day, the co-cultures were assembled, the medium containing IL-1β was substituted with fresh DMEM + 10% FBS containing 10 μg/mL of sEV or without sEV. All evaluations were carried out at 4 and 15 h after the addition of sEV. Chondrocytes and synoviocytes were used at passage 2 and 3, respectively.

### Real-time PCR

Cells from monocultures and cocultures previously treated with IL-1β and then kept in either CTR or sEV conditions were analyzed by Real-Time RT-PCR at 4 and 15 h to investigate the expression of IL-1β, IL-6, IL-8, MCP-1, TNF-α, MMP-1, MMP-10, ADAMTS4, ADAMTS5, MMP-13, COX-2, INOS and VEGF (Table 1). Total RNA was isolated using TRIZOL reagent (INVITROGEN) following the manufacturer’s recommended protocol^21^. The samples were then treated with DNase I (DNA-free Kit; AMBION, LIFE TECHNOLOGIES) and RNA quantified using Nanodrop spectrophotometer (EUROCLONE). The RNA was reverse transcribed using the SuperScript Vilo cDNA synthesis Kit (INVITROGEN), according to the manufacturer’s protocol. Real-Time PCR was run in a LightCycler Instrument (ROCHE MOLECULAR BIOCHEMICALS, Indianapolis, IN) using SYBR Premix Ex Taq (TAKARA, CLONTECH LABORATORIES, Mountain View, CA) with the following protocol: initial activation at 95 °C for 10 min, amplification for 45 cycles at 95° C for 5 s and at 60 °C for 20 s. mRNA levels were calculated for each target gene and normalized using the reference gene GAPDH according to the formula 2^−ΔCt^ and expressed as a percentage of the reference gene.

**Table 1:** List of primers used in Real-Time PCR.

RNA template	Primer sequences (forward:5′ and reverse:3′)	Annealing temperature (°C)
GAPDH	5′-TGGTATCGTGGAAGGACTCATGAC	60
	3′-ATGCCAGTGAGCTTCCCGTTCAGC	
IL-1β	5′-GTGGCAATGAGGATGACTTGTT	60
	3′-TGGTGGTCGGAGATTCGTAG	
IL-6	5′-TAGTGAGGAACAAGCCAGAG	60
	3′-GCGCAGAATGAGATGAGTTG	
IL-8	5′-CCAAACCTTTCCACCC	60
	3′-ACTTCTCCACAACCCT	
MCP-1	5′-GAAGCTCGCACTCTCGCCT	60
	3′-GAGTGTTCAAGTCTTCGGA	
TNF-α	5′-AGCCCATGTTGTAGCAAACC	60
	3′-ACCTGGGAGTAGATGAGGTA	
MMP-1	5′-TGGACCTGGAGGAAATCTTG	60
	3′-CCGCAACACGATGTAAGTTG	
MMP-10	5′-GCCAGTCCATGGAGCAAGGCT	60
	3′-TCGCCTAGCAATGTAACCAGCTGT	
ADAMTS4	5′-CTGCCTACAACCACCG	60
	3′-GCAACCAGAACCGTCC	
ADAMTS5	5′-GCACTTCAGCCACCATCAC	60
	3′-AGGCGAGCACAGACATCC	
MMP-13	5′-TCACGATGGCATTGCT	60
	3′-GCCGGTGTAGGTGTAGA	
COX-2	5′-CAGCACTTCACGCATCAGTTT	60
	3′-GCGCAGTTTACGCTGTCTA	
INOS	5′-ACATTGATCAGAAGCTGTCCCAC	60
	3′-AAAGGCTGTGAGTCCTGCAC	
VEGF	5′-TGATGATTCTGCCCTCCTC	60
	3′-GCCTTGCCTTGCTGCTC	

### Quantification of secreted factors

The supernatants collected from monocultures and co-cultures at 4 and 15 h were centrifuged to eliminate cellular debris and particulates and stored at − 80 °C until use. Successively, samples were evaluated for the release of IL-1β, IL-1ra, IL-2, IL-4, IL-6, IL-8, IL-9, IL-10, IL-12, IL-13, IL-15, Basic FGF, Eotaxin, G-CSF, GM-CSF, IFN-γ, IP-10, MCP-1, MIP-1α, MIP-1β, RANTES, TNF-α, and VEGF using Bio-Plex Protein Array System (BIO-RAD LABORATORIES, Hercules, CA and MILLIPORE CORPORATION, Billerica, MA) following the manufacturer’s instructions. Standard levels between 70 and 130% of the expected values were considered accurate and were used.

### Cellular uptake of sEV

Chondrocytes and synoviocytes were seeded in an 8 wells chamber slide at a density of 3 × 10^4^/cm^2^ for 3 days in DMEM (SIGMA-ALDRICH) with 10% FCS. sEV obtained from ADSC-CM were labeled with PKH26 (SIGMA-ALDRICH), according to the manufacturer's instructions. Briefly, sEV were incubated with PKH26 (4 μM) at RT for 10 min. Then, the labeled sEV were washed twice in PBS, centrifuged at 6000 × *g* for 10 min, resuspended in medium without sEV and incubated with chondrocytes and synoviocytes for 1, 2, 4, 6, 8, and 12 h at 37 °C. Just for imaging purpose and in order to enhance the signal of sEV uptake, a higher sEV concentration was used (100 µg/ml). Cells were thereafter fixed with 4% PFA, unmasked with either a solution of 0.02 U/mL of chondroitinase ABC in 50 mM TRIS–HCl pH 8 (for chondrocytes) or a solution of 0.1% of Triton X-100/PBS (for synoviocytes) and blocked with 4% BSA (SIGMA-ALDRICH) in 0.1% Triton X-100/TBS (dilution buffer, used for primary and secondary antibodies dilution) to avoid unspecific bindings. Successively, cells were incubated with 5 μg/mL of a mouse anti-human beta actin monoclonal antibody (SIGMA-ALDRICH), followed by incubation with 15 μg/mL of a donkey anti-mouse IgG Alexa Fluor 488 secondary antibody (THERMOFISHER SCIENTIFIC, Waltham, Massachusetts, USA). The nuclei were labeled with a DAPI solution (SIGMA-ALDRICH). Slides were mounted with a diazabicyclooctane-based antifade reagent (SIGMA-ALDRICH). Pictures were taken at high magnification with a NIKON A1-R confocal laser scanning microscope equipped with a NIKON 20×, 0.95 NA objective lens, and with 405, 488 and 561 nm laser lines to excite DAPI (blue), Alexa 488 (green) and PKH26 (red) fluorescence signals, respectively. Emission signals were detected by a photomultiplier tube (DU4) preceded by emission filters BP 525/50 nm and BP 595/50 nm for Alexa Fluor 488 and PKH26, respectively^22^. Laser scanning, image acquisition and processing were performed with NIKON Imaging Software NIS Elements AR-4 (NIKON Inc., USA). Optical sections were spaced *0.5 μm along the z axis and were digitized with a scanning mode format of 1024 × 1024 pixels and 4096 grey levels.

Additional experiments were carried out to support the reliability of the uptake imaging experiments. Due to the long aliphatic tail, PKH26 is prone to forming micelles of similar size to small EVs^23^. Therefore, to exclude that some signal could arise from micelles spontaneously originating from the dye, a labeling experiment was carried out using a ‘blank (the same PBS volume used to resuspend the purified sEV)’ that used the same concentration of PKH26 and underwent the same washing procedure. This control was applied to parallel cultures of chondrocytes and synoviocytes for 8 and 12 h at 37 °C.

### p65 immunofluorescence

Chondrocytes and synoviocytes were seeded in an 8 wells chamber slides at a density of 3 × 10^4^/cm^2^ cells for 3 days in DMEM (SIGMA-ALDRICH) with 10% FCS. Successively, cells were treated with IL-1β (5 ng/mL, R&D) for 18 h, then the medium containing IL-1β was removed and substituted in half of the wells with fresh DMEM + 10% FBS (CTR) and in the other half with the same medium containing 10 μg/mL of sEV and cultured for 4 and 15 h. At the end of each experimental time point (4 h and 15 h) the cells were fixed with 4% PFA, treated for antigen unmasking with a solution of 0.02 U/mL of chondroitinase ABC in 50 mM TRIS–HCl pH 8 (for chondrocytes) or with a solution of 0.1% of Triton X-100/PBS (for synoviocytes) and blocked with 4% bovine serum albumin (BSA) (SIGMA-ALDRICH) in 0.1% Triton X-100/TBS (dilution buffer, used for primary and secondary antibodies dilution) to avoid unspecific bindings. Successively, cells were incubated with rabbit anti-human p65 (ABCAM AB7970, rabbit polyclonal antibody, 5 µg/ml) for 4 h at RT, followed by incubation with 15 μg/mL of donkey anti-rabbit IgG Alexa Fluor 555 (THERMOFISHER SCIENTIFIC). The nuclei were labeled with a DAPI solution (SIGMA-ALDRICH). Slides were mounted with the antifade reagent and examined under the NIKON A1-R confocal laser scanning microscope as described above.

### Western blot analysis of NF-κB activation

The tuning of NF-κB signaling was further investigated by western blot analysis on both chondrocytes (2 × 10^5^) and synoviocytes (6 × 10^4^) plated in 24 well—plates in CTR (IL-1β stimulated) and sEV (IL-1β + sEV) conditions at both 4 and 15 h since sEV delivery. At the time of collection the medium was removed, the cells were recovered with a scraper using a small volume of cold PBS with the addition of inhibitors of phophatases and proteases. Then the cells were gently centrifuged and lysed with 20 µl of RIPA buffer. The samples were subsequently loaded, run and transferred to PVDF membranes as detailed previously.

Western blot was carried out with the following antibodies: Phospho-NF-κB p65(Ser536) (rabbit monoclonal antibody, clone 93H1, used at 1:1000, CELL SIGNALLING TECHNOLOGY #3033), and β-actin (mouse monoclonal, clone AC-74, used at 0.8 µg/ml SIGMA-ALDRICH # A2228) that served as loading control. Appropriate anti species HRP conjugated secondary antibodies were from JACKSON IMMUNORESEARCH.

### Statistical analysis

Data were expressed as mean and standard deviation of the mean (mean ± SD) and analyzed and graphed using the GraphPad Prism 5.0 software (GRAPHPAD SOFTWARE, La Jolla, CA, USA). Since comparisons were undertaken among multiple groups (CTR, sEV-treated at 4 and 15 h) ANOVA was used, followed by Tukey’s post hoc test. Again, the differences were considered significant and evidenced as *P < 0.05; **P < 0.01; and ***P < 0.001.

## Results

### sEV quantification and characterization

At the end of the isolation procedure starting from each 50 mL volume of ADSC-CM (several tubes with pooled supernatants were collected for each ADSC culture, established with an initial seeding of 2 × 10^3^ cells/cm^2^ and cultured to confluence from passage 2 to passage 4) a 200 µl volume of purified sEV was obtained.

**Figure 1:**
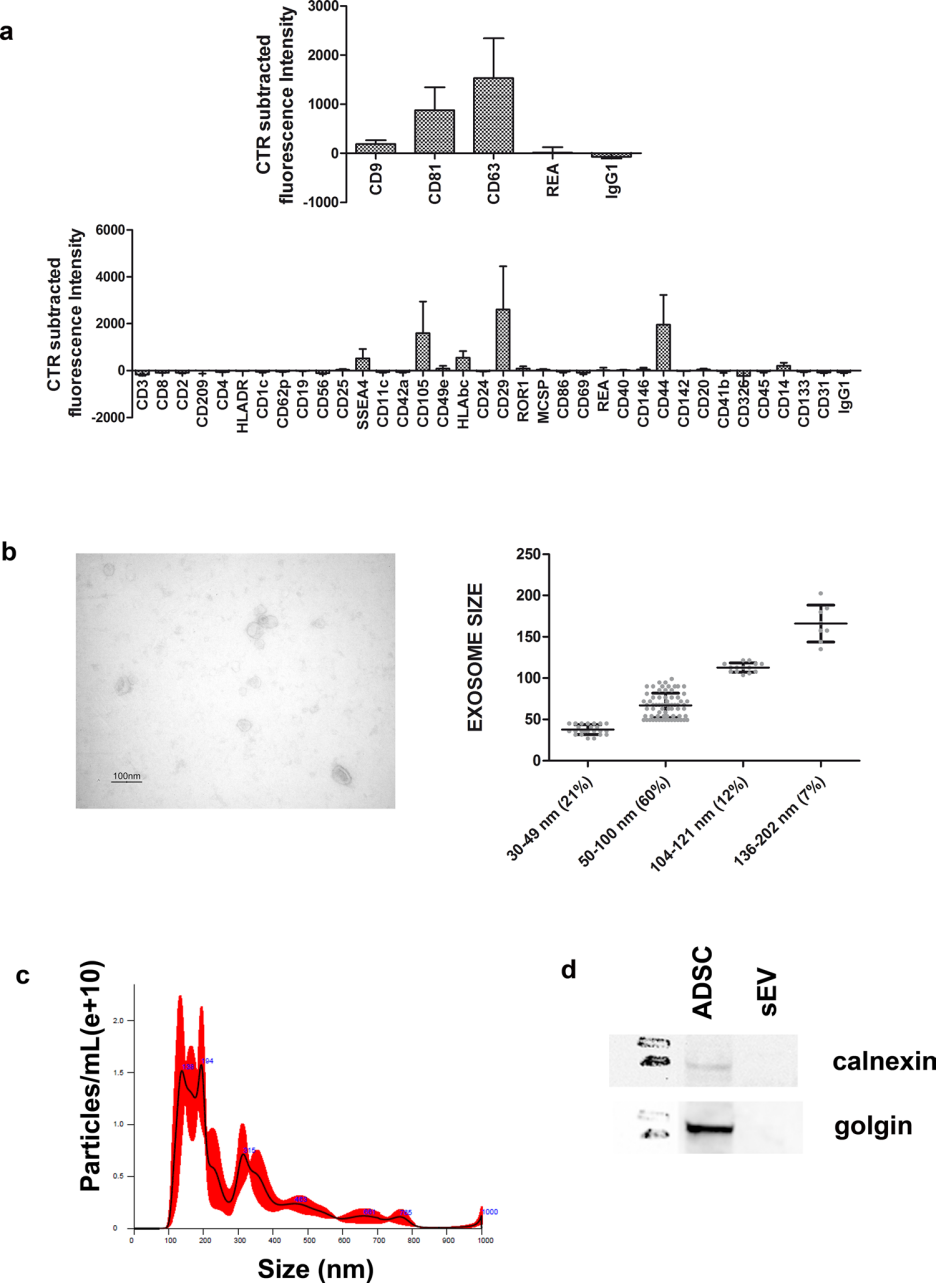
sEV characterization. (**a**) Surface Epitope characterization. Upper graph: control subtracted fluorescence intensity of CD9, CD63, CD81 markers and their corresponding isotype controls (IgG1, REA) of different purified sEV preparations (n = 3). Lower graph: control subtracted fluorescence intensity of the other 34 surface epitopes and isotype controls on the different purified sEV preparations (n = 3). (**b**) Transmission Electron Microscopy characterization. Left picture: transmission electron microscopy image of sEV isolated from ADSC supernatant. Scale bar, 100 nm. Right graph: data obtained from several sEV measured, grouped per size class with information about mean and standard deviation and percentage of each class. 60% of the sEV had a 50–100 nm size. (**c**) Nanotracker analysis reporting the average concentration/size. Error bars (red pattern) indicate + / − 1 standard error of the mean. (**d**) Western blot analysis indicating the absence of calnexin (marker of the endoplasmic reticulum) and of Golgin 97 (marker of Golgi apparatus) in the sEV samples (full blots available in supplementary Fig. 1).

The multiplex bead-based assay coupled with a flow cytometric analysis, confirmed that the sEV samples were positive for the specific sEV markers: CD63, a tetraspanin accumulating in multivesicular bodies, and CD9/CD81 that are localized mainly at the plasma membrane, even if with different intensity (Fig. 1a, upper graph). In particular, CD63 and CD81 expression was higher with respect to CD9. Moreover, sEV showed a strong positivity for the mesenchymal stromal markers CD29, CD44, CD105 and for the stage specific embryonic antigen-4 (SSEA-4) usually associated to cellular pluripotency. Details of all surface markers and isotypes are shown in Fig. 1a, lower graph.

By transmission electron microscopy, we found round or cup-shaped vesicles (Fig. 1b, left image) with a prevalent size of 50–100 nm in diameter (Fig. 1b, right graph, representing the distribution of vesicles in different size classes, with evidence of individual measurements together with means ± standard deviation).

The nanoparticle tracking assay of sEV at the end of the combined precipitation and size exclusion chromatography purification showed a distribution plot of particle hydrodynamic diameter consistent with extracellular vesicles, with a mode +/− Standard Error of size distribution of 152.4 +/− 10.4 nm. Particles concentration resulted of 2.78 × 10^10^ +/− 2.72 × 10^8^ particles/ml (Fig. 1c).

Western blot analysis of sEV along with 150,000 ADSC confirmed the absence of ADSC organelle contamination: Fig. 1d indicates no signal from either the endoplasmic reticulum (calnexin) or the Golgi apparatus (Golgin 97).

### sEV uptake

**Figure 2:**
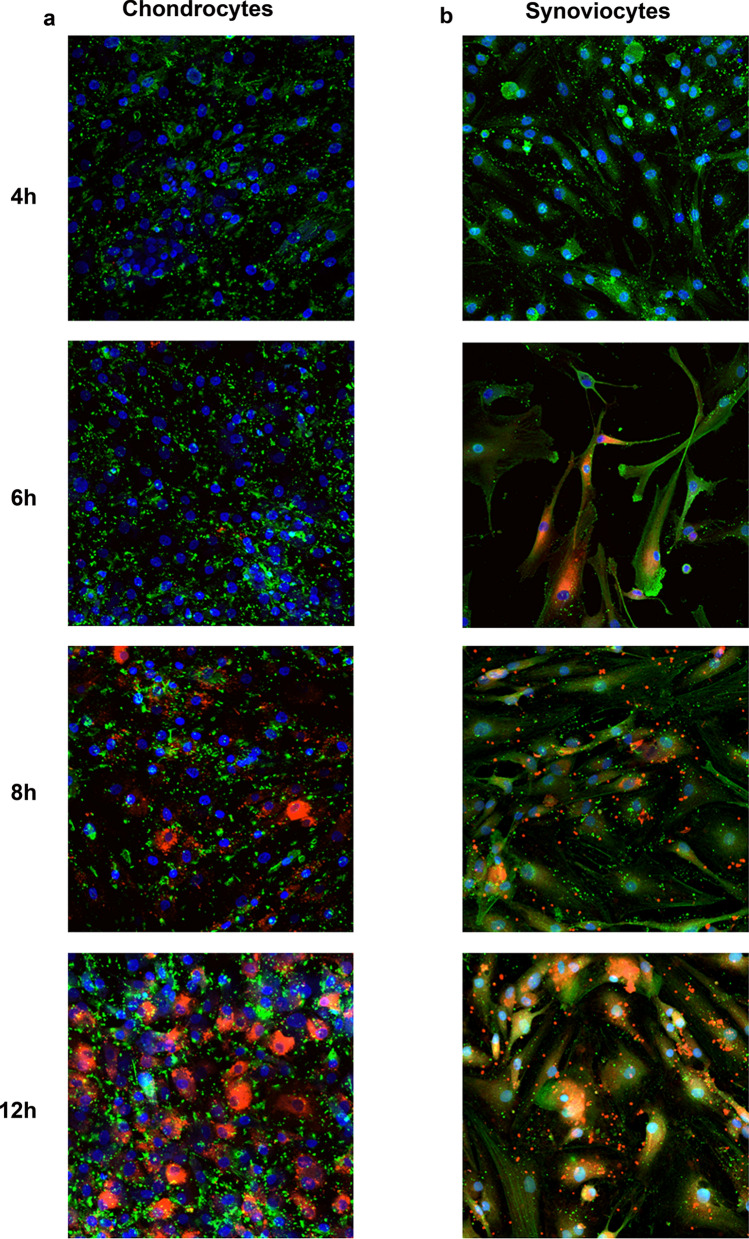
Cellular uptake assay at 4, 6, 8, and 12 h on chondrocytes (**a**) and synoviocytes (**b**). Merged images of PKH26-labelled sEV (red), cytoskeleton (green) and nuclei (blue) staining revealed the localization of sEV in the cytoplasm and around nuclei. (**a**) Chondrocyte and (**b**) synoviocyte representative images obtained with a NIKON A1-R confocal laser scanning microscope equipped with PlanApo VC 20 × Air 0.75NA objective lens.

To verify if ADSC derived sEV were able to directly interact with both chondrocytes and synoviocytes thus triggering functional changes, cells were incubated with PKH26 labeled sEV for 1, 2, 4, 6, 8 and 12 h. Cells took up sEV and became fluorescent with a different kinetic. Chondrocytes started to endocytose sEV after 8 h reaching their maximum fluorescent intensity at 12 h (Fig. 2a), while synoviocytes progressively internalized sEV from 6 h up to 12 h (Fig. 2b). In both cell types, labeled sEV were mainly localized in the cytoplasm and around the nucleus. Synoviocytes also showed, starting at 8 h, PKH26 labeled vesicles of variable size in the extracellular spaces or close to the plasma membrane, suggesting the secondary release of microvesicles from this cell type. For both chondrocytes and synoviocytes sEV internalization was not observed at 1, 2, and 4 h (1 and 2 h: data not shown). The delayed appearance of PKH26 labeled vesicles and the different kinetics shown by the two cell types argue in favor of the reliability of the findings and exclude biases arising from spontaneous micelle formation starting from PKH26^23^. Moreover, the control experiment carried out to exclude biases due to PKH26 spontaneous micelle formation confirms that this signal is negligible compared to that of PKH26 labeled sEV (supplementary Fig. 2).

### sEV effects on gene expression in chondrocytes and synoviocytes

In order to evaluate if sEV were able to down-regulate the expression of catabolic and inflammatory genes in both chondrocytes and synoviocytes, cells were pre-treated with IL-1β, a cytokine widely used in in vitro OA models, able to induce an inflammatory environment. The expression of several genes involved in OA pathophysiology was evaluated, including inflammatory cytokines/chemokines (IL-1β, IL-6, IL-8, MCP-1 and TNF-α), extracellular matrix degradative enzymes (MMP-1,-10, -13, ADAMTS4, and ADAMTS5), and molecules related to angiogenetic/pain processes such INOS, VEGF, and COX-2.

**Figure 3:**
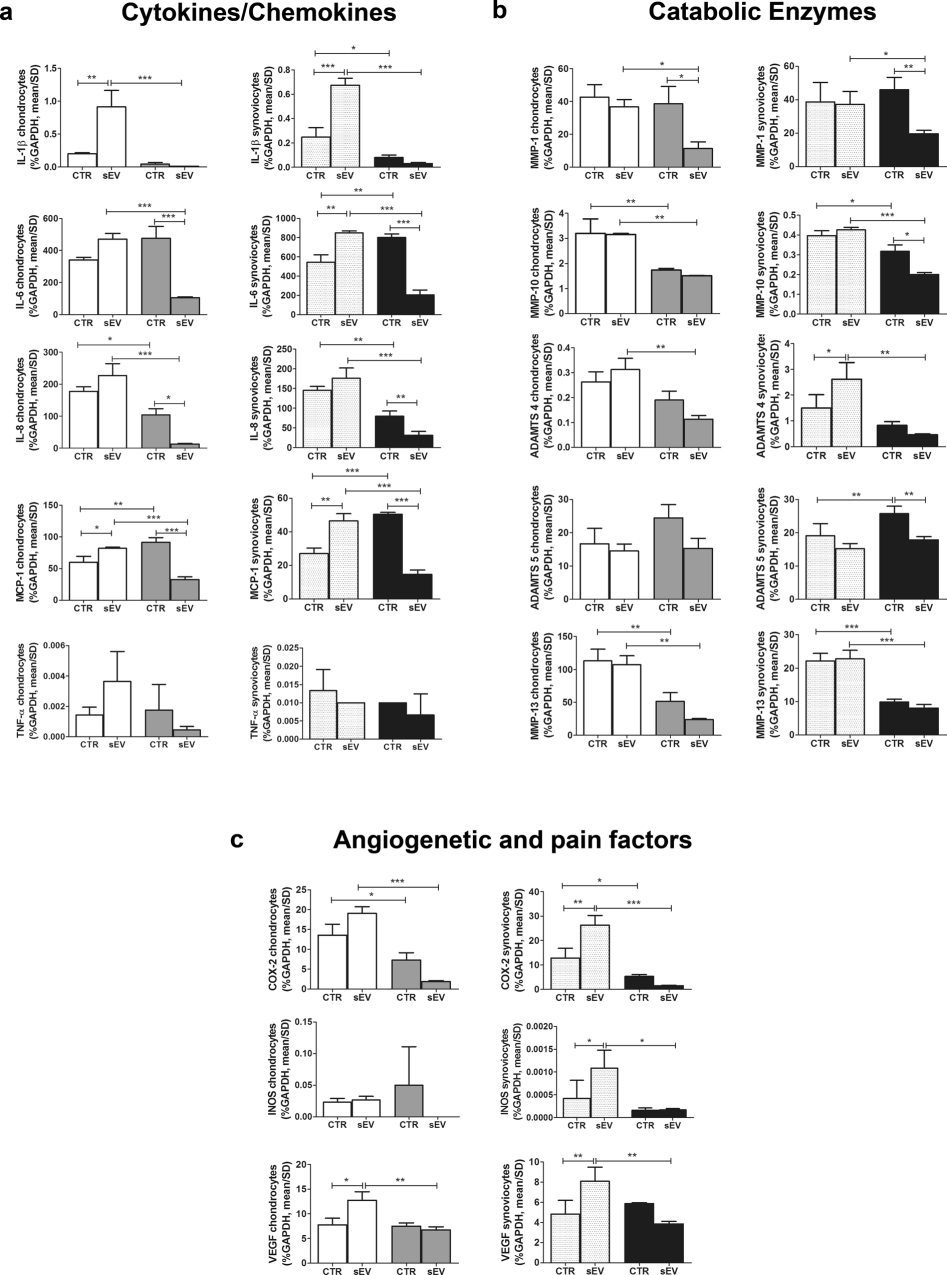
sEV effects on gene expression of Cytokines/Chemokines (**a**), Catabolic Enzymes (**b**) and Angiogenetic and Pain Factors (**c**). Data were normalized to GAPDH. Each graph reports data collected from 3 independent sEV samples and expressed as means ± SD. Data were compared by ANOVA, followed by Tukey’s post hoc test, with *p < 0.05, **p < 0.01, ***p < 0.001. Left graphs are related to chondrocytes while right graphs are related to synoviocytes. Different patterns are used for different cells and time points: chondrocytes at 4 h: white; chondrocytes at 15 h: gray; synoviocytes at 4 h: finely dotted; synoviocytes at 15 h: black.

In general, compared to the CTR, sEV induced a different gene expression, strictly related to the experimental time point. In chondrocytes, sEV significantly increased the expression of some cytokine/chemokine genes (IL-1β, MCP-1) and angiogenetic factors (VEGF) with respect to the CTR at 4 h. Conversely, at 15 h, the levels of some of these genes (IL-6, IL-8, MCP-1, MMP-1) were significantly reduced by the addition of sEV (Fig. 3).

Synoviocytes showed a similar trend: at 4 h, the presence of sEV induced a higher expression of cytokines/chemokines, catabolic enzymes and inflammatory genes (IL-1β/IL-6/MCP-1, ADAMTS4 and COX-2/INOS/VEGF respectively) compared to the CTR. On the contrary, at 15 h sEV markedly down regulated the expression of IL-6, IL-8, MCP-1, MMP-1, MMP-10 and ADAMTS5 (Fig. 3).

sEV addition on chondrocytes and synoviocytes grown in co-culture, which represents a more physiological cell environment somehow mimicking the articular environment, was not able to reproduce the above effects on gene expression in both chondrocytes and synoviocytes, with the notable exception of a significant reduction of ADAMTS5 in chondrocytes at 15 h (Supplementary Fig. 4).

**Figure 4:**
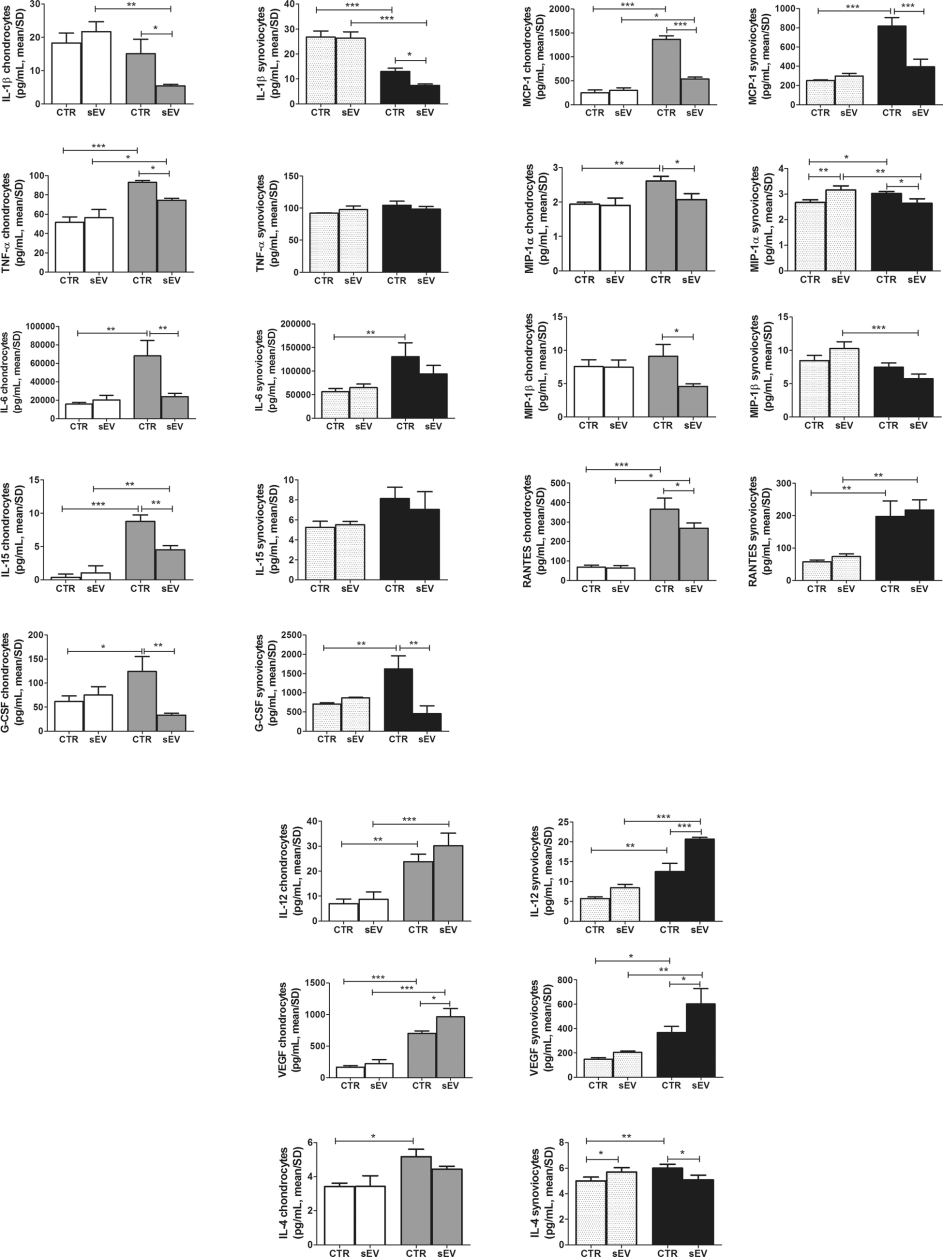
sEV effects on Cytokines/Chemokines protein release. Each graph reports data collected from 3 independent sEV samples and expressed as means ± SD. Data were compared by ANOVA, followed by Tukey’s post hoc test, with *p < 0.05, **p < 0.01, ***p < 0.001. Left graphs report the protein release from chondrocytes and right graphs that from synoviocytes. Different pattern are used for different cells and time points: chondrocytes at 4 h: white; chondrocytes at 15 h: gray; synoviocytes at 4 h: finely dotted; synoviocytes at 15 h: black.

### sEV effects on secreted factors

To validate if sEV were able to reduce the effects of IL-1β inflammatory stimulus, the protein release of a panel of cytokines/chemokines was investigated by Bioplex technology. Both supernatants obtained from monocultures and co-cultures were evaluated.

In monocultures, sEV confirmed their differential effects depending on the experimental time scheduled. The relevant results are shown in Fig. 4, while findings about other soluble factors not significantly affected by sEV are presented in Supplementary Fig. 3.

On chondrocytes, at 4 h there were no significant differences between sEV and CTR groups. At 15 h instead, sEV induced a significantly lower release of IL-1β, TNF-α, IL-6, IL-15, G-CSF, MCP-1, MIP-1α, MIP-1β and RANTES respect to the CTR, while VEGF was upregulated (Fig. 4).

On synoviocytes, sEV stimulated a significantly higher production of MIP-1α and IL-4, compared to CTR at 4 h (Fig. 4), while for the other molecules there was no statistically significant difference between the two groups. Conversely, at 15 h sEV induced a significantly lower release of IL-1β, G-CSF, MCP-1, MIP-1α and IL-4 respect to the CTR group (Fig. 4). Among the inflammatory molecules, only IL-12 and VEGF were increased in the sEV group respect to CTR at 15 h.

The analysis of supernatants derived from the co-cultures again showed differential effects of sEV delivery at either 4 or 15 h. At 4 h sEV significantly increased the release of MCP-1 and MIP-1β, while at 15 h, sEV induced a significantly lower release of MIP-1α, MIP-1β, RANTES and G-CSF, compared to CTR, while for the other molecules analyzed there was no statistically significant difference between the two groups (Supplementary Fig. 5).

**Figure 5:**
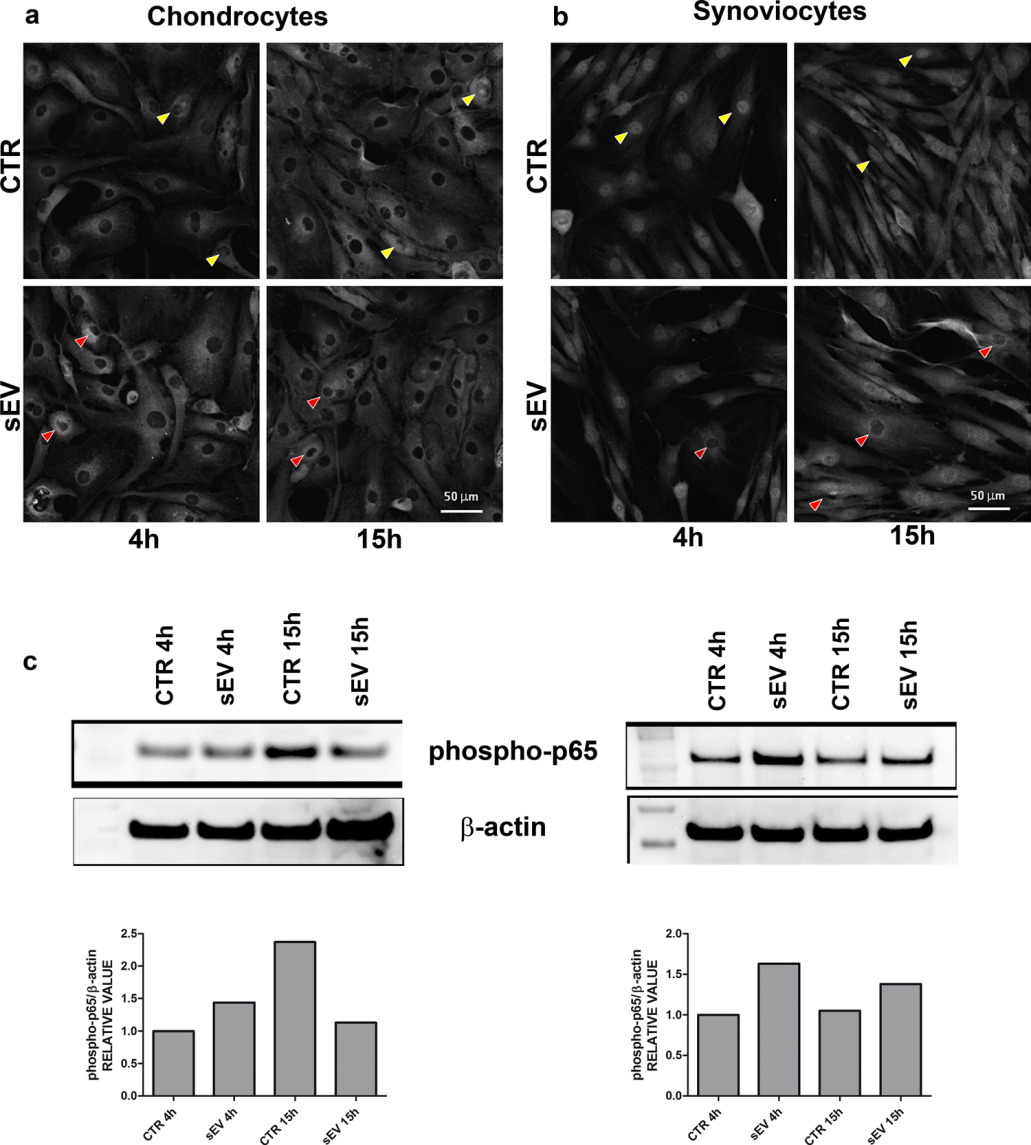
p65 immunofluorescence on chondrocytes (**a**) and synoviocytes (**b**) at 4 and 15 h in CTR (IL-1β treated) and sEV (IL-1β + sEV treated) conditions. Scale bar: 50 μm. Yellow arrows: p65 nuclear localization has a marked different pattern in chondrocytes and synoviocytes. Red arrows: p65 localization mainly excluded from the nuclei in some chondrocytes and synoviocytes at 4 and 15 h following sEV addition to cultures previously treated with IL-1β. (**c**) Western blot analysis of samples of chondrocytes (left) and synoviocytes (right) treated as those shown in (**a**) and (**b**), but dedicated to western blot analysis of phosphorylation of p65 and β actin as loading control (full blots available in supplementary Figs. 6 and 7) Lower graphs: relative quantification of phospho-p65 signal normalized to that of β-actin.

### sEV effects on p65

To explore whether sEV were able to inhibit the activation of the NF-κB signaling pathway, the cytoplasm to nucleus translocation of RelA (p65) was investigated, a protein contained in the NF-κB heterodimers involved in the activation of most of the genes listed above. In chondrocytes, there was a prevalent cytoplasmic accumulation of p65 in both CTR (IL-1β treated) and sEV (IL-1β treated + sEV treated) group, with only a few nuclei showing a discrete p65 signal appearing as discrete spots or fibrillar staining. This appeared slightly more evident in the CTR group, while in the sEV group an increased perinuclear p65 signal was evident close to the nuclear membrane, suggesting an active p65 exclusion from the nucleus, as early as 4 h (Fig. 5a). Conversely, in synoviocytes p65 was prevalently localized into the nuclei of CTR (IL-1β treated) cells particularly at 4 h, with a strong and diffuse nuclear staining, while in sEV (IL-1β treated + sEV treated) group a decreased p65 nuclear staining was observed mostly at 15 h, implying that sEV were effective in either reducing p65 translocation to the nucleus or in triggering its return and maintenance in the cytoplasm (Fig. 5b). Western blot analysis of parallel samples confirmed a reduced phosphorylation of p65 at 15 h in chondrocytes treated with sEV, while in synoviocytes addition of sEV resulted in an immediate slight increase in phospho-p65 signaling, in keeping with early increased expression of NF-κB target genes, while at 15 h CTR and sEV samples were similar (Fig. 5c). It is conceivable that the early enhancement of NF-κB includes increase of IκBα transcription and expression^24^, with maintenance of p65 in its cytoplasmic location.

## Discussion

MSC have been recently renamed as “Medicinal Signaling Cells” because of their powerful paracrine activity^25^, that includes both trophic and immunomodulatory effects. Due to the higher yield and ease of collection, most clinical work particularly in the field of OA has recently focused on the use of ADSC. Encouraging clinical results have elicited several in vitro and/or in vivo studies to elucidate the molecular and biomolecular basis of these beneficial effects^26^. A growing attention is dedicated to the analysis of the functional effects of the different types of secreted extracellular vesicles. In most cases, microvesicles and EXO differ with regards to their biogenesis, size and mechanisms of uptake, although current state of art suggests that vesicles cannot be efficiently separated^15^. Compared to the few available papers, which mainly used differential centrifugation to collect sEV, in this study a combination of precipitation and size exclusion chromatography was included to ensure efficient removal of larger vesicles, allowing the purification of a rather homogeneously sized vesicle population, according to electron microscopy and NTA analysis. Thus, this study documented the effects of ADSC derived purified sEV on two cell types involved in the pathogenesis of OA.

Chondrocytes and synoviocytes, specifically primary human cells derived from OA patients, have been investigated. To study the kinetic of sEV uptake and downstream functional effects, chondrocytes and synoviocytes have been evaluated and kept in culture models that best maintain their differentiated phenotype. These cells have been tested both separately and in co-culture, to reproduce the crosstalk that enhances the inflammatory loops in an OA joint. The cellular uptake of labeled sEV was evaluated on both cell types across a wide time window (1–12 h) exploiting confocal microscopy, thus disclosing peculiar features in the behavior of these cells. Both chondrocytes and synoviocytes showed evidence of intracellular accumulation of sEV, that were found to occupy the areas closer to the nuclear membrane. More in detail, chondrocytes with evidence of intracellular sEV were only a sporadic finding prior to 8 h, while at 8 and 12 h the majority of chondrocytes showed large amounts of intracellular sEV. On the other hand, synoviocytes showed a progressively increasing uptake, as earlier as 6 h, and markedly increased at later time points, in line with previous findings^27^. The different uptake pattern suggests more anticipated/marked sEV effects on synoviocytes compared to chondrocytes, in agreement with the findings of a more robust sEV functional activity on synoviocytes with regards to gene expression modulation and effects on the NF-κB signaling pathway. Furthermore, imaging showed a peculiar sEV activity on synoviocytes not observed in chondrocytes: as early as 8 h, images of new vesicles were evident among cells, suggesting a secondary release from synoviocytes, confirming a behavior previously reported at 24 h of incubation^27^ with labeled vesicles. This likely suggests the possibility of positive feedback loops that enhance the trophic activities of ADSC derived sEV. Noteworthy, the size of the observed newly released vesicles is quite heterogeneous, indicating synoviocyte release of both microvesicles and sEV.

Beside the kinetics, also the effects of sEV on the two cell types differed. In this study, the ability of sEV to attenuate inflammation was tested. To this aim, IL-1β was delivered, as OA target gene validated by functional genomics in the DMM model^28^. In this setting, sEV effects at the level of gene and protein expression were evaluated at two time points: 4 and 15 h. In particular, sEV were evaluated for their ability to tune IL-1β induction of genes belonging to the following families: 1) soluble inflammatory mediators such as cytokines and chemokines, 2) catabolic enzymes and 3) angiogenetic and pain related genes. Most of these genes were also assessed at the protein level by means of a bead-based sandwich immunoassay technology with high sensitivity and dynamic range.

**Figure 6:**
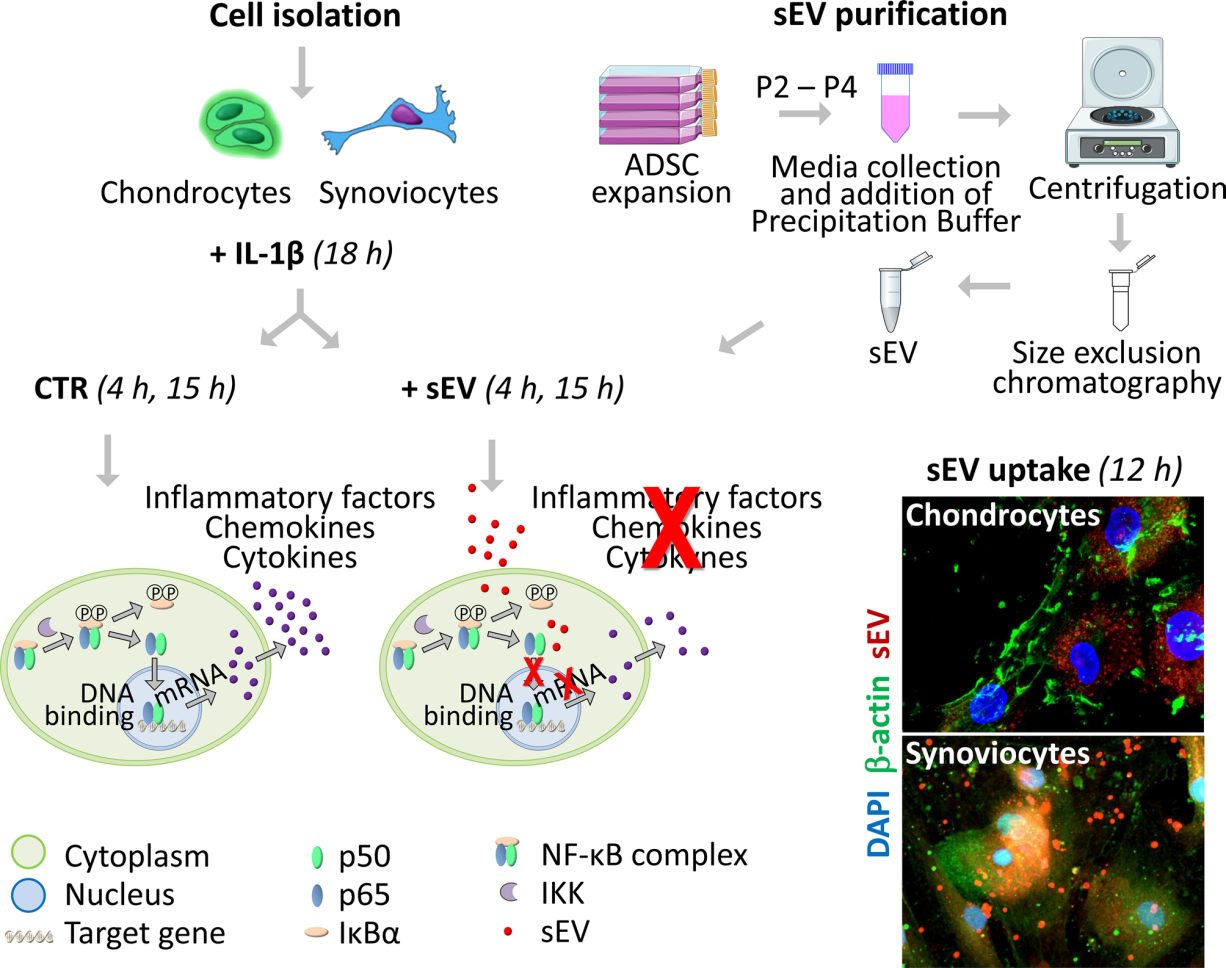
Experimental outline of the research work undertaken in the paper, from sEV purification to sEV addition to primary cultures of chondrocytes and synoviocytes previously treated with IL-1β. Molecular hypothesis underlying the observed effects.

The overall experimental procedure is outlined in Fig. 6.

Previous papers focused on the effects of either microvesicles or EXO on chondrocytes and cartilage explants^29^ or on the effects of a mixture of these vesicles on synovial fibroblasts^27^ at 24 h. While this time point could represent a good compromise to look at both gene and protein expression, an earlier kinetics allowed to tease out peculiar details.

In this study, in most cases at the earlier time point (4 h), particularly in synoviocytes, sEV addition resulted in an overall enhanced gene expression compared to CTR. These adaptations occur when sEVs are not yet internalized inside target cells suggesting that ADSC-derived vesicles could act triggering synoviocytes/chondrocytes via direct interaction of sEV CD44 with pericellular hyaluronan. On the other hand, in most cases at 15 h when sEVs have been fully taken up, an evident reduction in inflammation markers was documented in sEV-treated samples. This suggests that the delivery of sEV cargo can impact intracellular signaling pathways^30^, in the end turning off inflammation in either chondrocytes (C) or synoviocytes (S), or both. Recent findings have pointed at sEV ability to counteract NF-κB by epigenetic mechanisms^31^. Accordingly, comparing CTR (IL-1β treated) and sEV (IL-1β + sEV) cells at 15 h, a significant reduction was observed in gene expression of IL-6 (C, S), IL-8 (C, S), MCP-1 (C, S), MMP-1 (C,S), MMP-10 (S) and ADAMTS5 (S). These findings were confirmed at the protein level, with significant reduction of the release of IL-1β (C,S), TNFα (C), IL-6 (C), IL-15 (C), G-CSF (C,S), MCP-1 (C,S), MIP-1α (C,S), MIP-1β (C), RANTES (C) and IL-4 (S). Among sEV samples, a dramatic reduction was observed between gene expression at 4 h compared to gene expression at 15 h for IL-1β, IL-6, IL-8 and MCP-1 for both cell types. It is interesting to underline that reduction of IL-1β expression indicates that sEV have the potential to counteract a pivotal inflammatory factor responsible for enhancing inflammation. All these cytokines are NF-κB target genes, whose induction has been referred to promoter activation by binding of NF-κB heterodimers that include RelA, i.e. p65. Previous papers started to investigate sEV effects on major signaling pathways within OA related models either in vitro or in vivo: AKT and ERK activation downstream delivery of MSC derived EXO to rat chondrocytes was assessed across a 0–12 h kinetic^32^, while tuning of NF-κB activation was shown at shorter time points, i.e. within 1 h post EXO or EV delivery in human chondrocytes^29^. Other papers highlighted the ability of EXO derived from either ADSC^33^ or different sources of stem/stromal cells^34^ to inhibit NF-κB activation in brain cells, as indicated by reduced nuclear p65 signal.

This study specifically addressed the effects of ADSC derived sEV on NF-κB activation in chondrocytes and synoviocytes. It is worth underlining that the use of cells within the 2nd, 3rd passage allowed to deal with synoviocytes retaining both synovial fibroblasts and synovial macrophages^35^. sEV delivery effects on p65 nuclear localization were investigated, focusing on the kinetic of sEV tuning of NF-κB activation, as evaluated by mean of nuclear to cytoplasm translocation of p65. In CTR conditions (i.e. IL-1β treated cells) and at the early time point (4 h), the pattern of p65 signal and its nuclear/cytoplasmic ratio was quite different in chondrocytes and synoviocytes: p65 was more cytoplasmatic in chondrocytes, with evidence of faint nuclear signals in some cells, while p65 was much more nuclear in synoviocytes, which exhibited a diffuse and strong nuclear signal. sEV administration affected more significantly the synoviocytes according to the localization of p65: an evident pattern of nuclear p65 exclusion was appreciated in synoviocytes as early as 4 h and even more frequently at 15 h, while in chondrocytes the increased p65 nuclear exclusion was suggested by a stronger p65 signal localized out of the nuclear membrane and evident as early as 4 h. In both chondrocytes and synoviocytes, western blot analysis showed increased p65 phosphorylation (about 1.5 fold increase) at 4 h, consistent with a further enhancement of NF-κB signaling responsible for a transient increase of NF-κB target genes at early time points (IL-1β, MCP-1 in both C and S; IL-6, ADAMTS4, COX-2 and iNOS in S). It is conceivable that this leads to an enhancement of IκBα transcription and translation being IκBα itself a NF-κB target gene, thus leading to the maintenance of cytoplasmic location of p65 at later time points^24^.

sEV ability to inhibit NF-κB activation is of great importance in OA management, given the pivotal role played by this signaling pathway in the disease^36^. Other relevant effects are those exerted on the gene expression of catabolic enzymes, namely the collagenase MMP-1 (C,S), and the stromelysin MMP-10 (S), an enzyme involved in the proteolytic, activating cleavage of collagenases^37^. MMP-10 is also responsible for activation of MMP-13, the key collagenase in OA^38,39^. Moreover, sEV have the ability to downregulate ADAMTS5, another OA target gene^28^, in synoviocytes. Another interesting family is that of genes involved in pain, i.e. COX-2, the inducible form of cyclooxygenase responsible for the production of prostaglandins, which mediate pain and inflammation. This enzyme, target of most anti-inflammatory drugs, appeared to be significantly modulated by sEV at 15 h (C,S) compared to the level at 4 h. Finally, the expression of VEGF, a factor involved in neo angiogenesis and in inflammation, that is strongly expressed in synovial lining cells in OA patients^40,41^, was also significantly downregulated by sEV at 15 h in both chondrocytes and synoviocytes compared to their levels at 4 h.

Among other genes, ADSC derived sEV showed the ability to inhibit MCP-1 expression at both gene and protein expression in both cell types, and this is relevant in the context of OA. Cumulative findings derived from OA patients and from OA animal models indicate the pivotal role played by this chemokine in OA: the severity of human OA as assessed by the radiological score correlates with MCP-1 level in the synovial fluid^42^ while serum level of MCP-1 correlates with pain score^43^ and radiographic progression^44^. Moreover, an early increase in MCP-1 has been reported following 2 surgical models of OA, the DMM^45^ and the rat meniscal tear model^46^. This is a major chemokine involved in macrophage recruitment, inflammation, and cartilage destruction^47^.

Most of the molecules involved in the OA process were significantly affected by sEV in both chondrocyte and synoviocyte cultures. On the other hand, less significant findings were obtained in the co-culture model, which could be explained by the lower ratio between the administered sEV and the cells. Dose kinetic experiments should be performed in order to find a dosage more suitable to elicit an appreciable effect also in the co-culture condition. Upon IL-1β stimulation, cells in co-culture release a large number of inflammatory molecules that further boost inflammation. This notwithstanding, a notable result was the reduced ADAMTS5 gene expression in chondrocytes at 15 h and reduced supernatant levels of MIP-α, MIP-1β, RANTES and G-CSF at 15 h. In this setting, it is likely that a higher sEV amount would be required to more clearly appreciate IL-1β counteracting effects. Available literature findings indeed indicate that the level of vesicles incorporation increases with time, reaching a maximum at 24 h, as well as with the vesicles/cell ratio up to a maximum of 100,000 vesicles/cell^27^. Future work is needed to find the optimal dosage to counteract inflammation in co-cultures of chondrocytes and synoviocytes, both kept at high density, a culture model that satisfactorily reproduces the OA joint environment^48^. While further co-culture studies are advisable, this study allowed to underline important findings in monocultures, both in terms of the effects on chondrocytes and synoviocytes, as well as to provide insights into sEV mechanisms modulating the inflammatory/catabolic OA environment.

## Conclusions

This study underlined the potential of ADSC derived sEV to affect gene expression and protein release of both chondrocytes and synoviocytes, counteracting IL-1β induced inflammatory effects, and provided insights into their mechanisms of action. sEV uptake was faster in synoviocytes, where it also elicited stronger effects, especially in terms of cytokine and chemokine modulation. The inflammatory/catabolic environment mediated by the NF-κB pathway was significantly attenuated by sEV, which hold promise as a new therapeutic strategy to address OA.

## Supplementary Information

Supplementary Information.

## Data Availability

The datasets generated during and/or analysed during the current study are available from the corresponding author on reasonable request.

## References

[1] Jafarzadeh SR, Felson DT (2018). Updated estimates suggest a much higher prevalence of arthritis in United States adults than previous ones. Arthritis Rheumatol..

[2] Aigner T, Rose J, Martin J, Buckwalter J (2004). Aging theories of primary osteoarthritis: From epidemiology to molecular biology. Rejuvenation Res..

[3] van der Kraan PM, van den Berg WB (2008). Osteoarthritis in the context of ageing and evolution. Loss of chondrocyte differentiation block during ageing. Ageing Res. Rev..

[4] Nuesch E (2011). All cause and disease specific mortality in patients with knee or hip osteoarthritis: Population based cohort study. BMJ.

[5] Wallace IJ (2017). Knee osteoarthritis has doubled in prevalence since the mid-20th century. Proc. Natl. Acad. Sci. USA.

[6] Cisternas MG (2009). Ambulatory visit utilization in a national, population-based sample of adults with osteoarthritis. Arthritis Rheum..

[7] Bayliss LE (2017). The effect of patient age at intervention on risk of implant revision after total replacement of the hip or knee: a population-based cohort study. Lancet.

[8] Deshpande BR (2016). Number of persons with symptomatic knee osteoarthritis in the US: Impact of race and ethnicity, age, sex, and obesity. Arthritis Care Res Hoboken.

[9] de Girolamo L (2016). Regenerative approaches for the treatment of early OA. Knee Surg. Sports Traumatol. Arthrosc..

[10] Filardo G (2016). Non-surgical treatments for the management of early osteoarthritis. Knee Surg. Sports Traumatol. Arthrosc..

[11] Filardo G (2013). Mesenchymal stem cells for the treatment of cartilage lesions: From preclinical findings to clinical application in orthopaedics. Knee Surg. Sports Traumatol. Arthrosc..

[12] Filardo G, Perdisa F, Roffi A, Marcacci M, Kon E (2016). Stem cells in articular cartilage regeneration. J. Orthop. Surg. Res..

[13] D’Arrigo D (2019). Secretome and extracellular vesicles as new biological therapies for knee osteoarthritis: A systematic review. J. Clin. Med..

[14] Madrigal M, Rao KS, Riordan NH (2014). A review of therapeutic effects of mesenchymal stem cell secretions and induction of secretory modification by different culture methods. J. Transl. Med..

[15] Mathieu M, Martin-Jaular L, Lavieu G, Thery C (2019). Specificities of secretion and uptake of exosomes and other extracellular vesicles for cell-to-cell communication. Nat. Cell Biol..

[16] Valadi H (2007). Exosome-mediated transfer of mRNAs and microRNAs is a novel mechanism of genetic exchange between cells. Nat. Cell Biol..

[17] Thery C (2018). Minimal information for studies of extracellular vesicles 2018 (MISEV2018): A position statement of the International Society for Extracellular Vesicles and update of the MISEV2014 guidelines. J. Extracell Ves..

[18] Guescini M (2017). Extracellular vesicles released by oxidatively injured or intact C2C12 myotubes promote distinct responses converging toward myogenesis. Int. J. Mol. Sci..

[19] Perut F, Roncuzzi L, Zini N, Massa A, Baldini N (2019). Extracellular nanovesicles secreted by human osteosarcoma cells promote angiogenesis. Cancers Basel.

[20] Minguzzi M (2019). Polyamine supplementation reduces DNA damage in adipose stem cells cultured in 3-D. Sci. Rep..

[21] Roffi A (2014). Does platelet-rich plasma freeze-thawing influence growth factor release and their effects on chondrocytes and synoviocytes?. Biomed. Res. Int..

[22] Assirelli E (2014). Human osteoarthritic cartilage shows reduced in vivo expression of IL-4, a chondroprotective cytokine that differentially modulates IL-1beta-stimulated production of chemokines and matrix-degrading enzymes in vitro. PLoS ONE.

[23] Puzar Dominkus P (2018). PKH26 labeling of extracellular vesicles: Characterization and cellular internalization of contaminating PKH26 nanoparticles. Biochim. Biophys. Acta Biomembr..

[24] Oeckinghaus A, Ghosh S (2009). The NF-kappaB family of transcription factors and its regulation. Cold Spring Harb. Perspect. Biol..

[25] Caplan AI (2017). Mesenchymal stem cells: Time to change the name!. Stem Cells Transl. Med..

[26] Perdisa F (2015). Adipose-derived mesenchymal stem cells for the treatment of articular cartilage: A systematic review on preclinical and clinical evidence. Stem Cells Int..

[27] Ragni E (2019). Interaction with hyaluronan matrix and miRNA cargo as contributors for in vitro potential of mesenchymal stem cell-derived extracellular vesicles in a model of human osteoarthritic synoviocytes. Stem Cell Res. Ther..

[28] Glasson SS (2007). In vivo osteoarthritis target validation utilizing genetically-modified mice. Curr. Drug Targets.

[29] Tofino-Vian M, Guillen MI, Perez Del Caz MD, Silvestre A, Alcaraz MJ (2018). Microvesicles from human adipose tissue-derived mesenchymal stem cells as a new protective strategy in osteoarthritic chondrocytes. Cell Physiol. Biochem..

[30] Mathivanan S, Ji H, Simpson RJ (2010). Exosomes: Extracellular organelles important in intercellular communication. J. Proteomics.

[31] Qiu B, Xu X, Yi P, Hao Y (2020). Curcumin reinforces MSC-derived exosomes in attenuating osteoarthritis via modulating the miR-124/NF-kB and miR-143/ROCK1/TLR9 signalling pathways. J. Cell Mol. Med..

[32] Zhang S (2018). MSC exosomes mediate cartilage repair by enhancing proliferation, attenuating apoptosis and modulating immune reactivity. Biomaterials.

[33] Feng N, Jia Y, Huang X (2019). Exosomes from adipose-derived stem cells alleviate neural injury caused by microglia activation via suppressing NF-kB and MAPK pathway. J. Neuroimmunol..

[34] Wang L (2018). Mesenchymal stem cell-derived exosomes reduce A1 astrocytes via downregulation of phosphorylated NFkappaB P65 subunit in spinal cord injury. Cell Physiol. Biochem..

[35] Manferdini C (2016). From osteoarthritic synovium to synovial-derived cells characterization: Synovial macrophages are key effector cells. Arthritis Res. Ther..

[36] Marcu KB, Otero M, Olivotto E, Borzi RM, Goldring MB (2010). NF-kappaB signaling: Multiple angles to target OA. Curr. Drug Targets.

[37] Barksby HE (2006). Matrix metalloproteinase 10 promotion of collagenolysis via procollagenase activation: Implications for cartilage degradation in arthritis. Arthritis Rheum..

[38] Goldring MB (2011). Roles of inflammatory and anabolic cytokines in cartilage metabolism: Signals and multiple effectors converge upon MMP-13 regulation in osteoarthritis. Eur. Cell Mater..

[39] Olivotto E (2013). IKKalpha/CHUK regulates extracellular matrix remodeling independent of its kinase activity to facilitate articular chondrocyte differentiation. PLoS ONE.

[40] Giatromanolaki A (2001). The angiogenic pathway "vascular endothelial growth factor/flk-1(KDR)-receptor" in rheumatoid arthritis and osteoarthritis. J. Pathol..

[41] Hamilton JL (2016). Targeting VEGF and its receptors for the treatment of osteoarthritis and associated pain. J. Bone Miner Res..

[42] Scanzello CR, Goldring SR (2012). The role of synovitis in osteoarthritis pathogenesis. Bone.

[43] Li L, Jiang BE (2015). Serum and synovial fluid chemokine ligand 2/monocyte chemoattractant protein 1 concentrations correlates with symptomatic severity in patients with knee osteoarthritis. Ann. Clin. Biochem.

[44] Longobardi L (2018). Associations between the chemokine biomarker CCL2 and knee osteoarthritis outcomes: The Johnston County Osteoarthritis Project. Osteoarth. Cartil..

[45] Miotla Zarebska J (2017). CCL2 and CCR2 regulate pain-related behaviour and early gene expression in post-traumatic murine osteoarthritis but contribute little to chondropathy. Osteoarth. Cartil..

[46] Wei T (2010). Analysis of early changes in the articular cartilage transcriptisome in the rat meniscal tear model of osteoarthritis: Pathway comparisons with the rat anterior cruciate transection model and with human osteoarthritic cartilage. Osteoarth. Cartil..

[47] Raghu H (2017). CCL2/CCR2, but not CCL5/CCR5, mediates monocyte recruitment, inflammation and cartilage destruction in osteoarthritis. Ann. Rheum. Dis..

[48] Pagani S (2019). The *N*-acetyl phenylalanine glucosamine derivative attenuates the inflammatory/catabolic environment in a chondrocyte-synoviocyte co-culture system. Sci. Rep..

