# Supplementary material for: Small Extracellular Vesicles from adipose derived stromal cells significantly attenuate in vitro the NF-κB dependent inflammatory/catabolic environment of osteoarthritis
Source: Sci Rep. 2021 Jan 13;11:1053. doi: 10.1038/s41598-020-80032-7 (PMC7806716; doi:10.1038/s41598-020-80032-7)
Supplement: Supplementary file 1 — Supplementary Information. [file 41598_2020_80032_MOESM1_ESM.pdf]

SUPPLEMENTARY MATERIAL

Small Extracellular Vesicles from Adipose Derived Stromal Cells significantly attenuate *in vitro* the NF-κB dependent inflammatory/catabolic environment of Osteoarthritis

Carola Cavallo[1], Giulia Merli[2], Rosa Maria Borzì[3,*], Nicoletta Zini[4,5], Stefania D'Adamo[6], Michele Guescini[7], Brunella Grigolo[1], Alessandro Di Martino[8], Spartaco Santi [4,5,#] and Giuseppe Filardo[2,#].

[1] Laboratorio RAMSES, IRCCS Istituto Ortopedico Rizzoli, via di Barbiano 1/10, 40136, Bologna, Italy.

[2] Applied and Translational Research center (ATRc), IRCCS Istituto Ortopedico Rizzoli, via di Barbiano 1/10, 40136, Bologna, Italy.

[3] Laboratorio di Immunoreumatologia e Rigenerazione tissutale, IRCCS Istituto Ortopedico Rizzoli, via di Barbiano 1/10, 40136, Bologna, Italy.

[4] CNR Institute of Molecular Genetics "Luigi Luca Cavalli-Sforza", Unit of Bologna, Via di Barbiano 1/10, 40136, Bologna, Italy.

[5] IRCCS, Istituto Ortopedico Rizzoli, Via di Barbiano 1/10, 40136, Bologna, Italy.

[6] Department of Medical and Surgical Sciences, University of Bologna, Bologna, Italy.

[7] Department of Biomolecular Science, University of Urbino Carlo Bo, Urbino, Italy.

[8] Clinica Ortopedica e Traumatologica 2, IRCCS Istituto Ortopedico Rizzoli, Bologna, Italy.

***** Correspondence**:** E-mail: rosamaria.borzi@ior.it;

\# These authors contributed equally to this work

S1

A

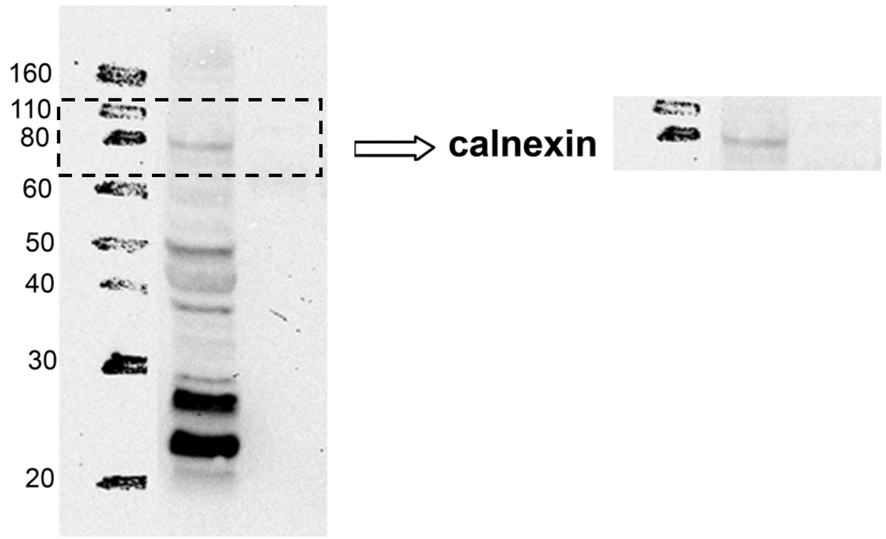
160
110
80
60
50
40
30
20
calnexin

B

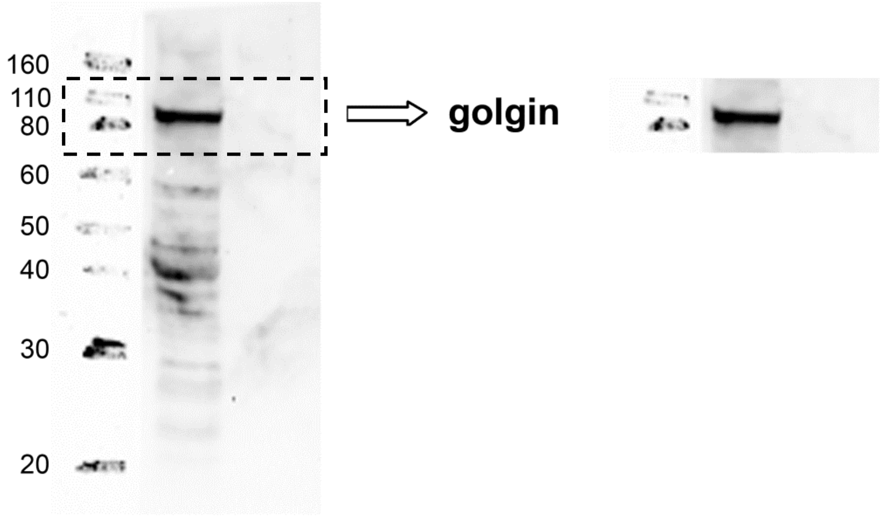
160
110
80
60
50
40
30
20
golgin

S2

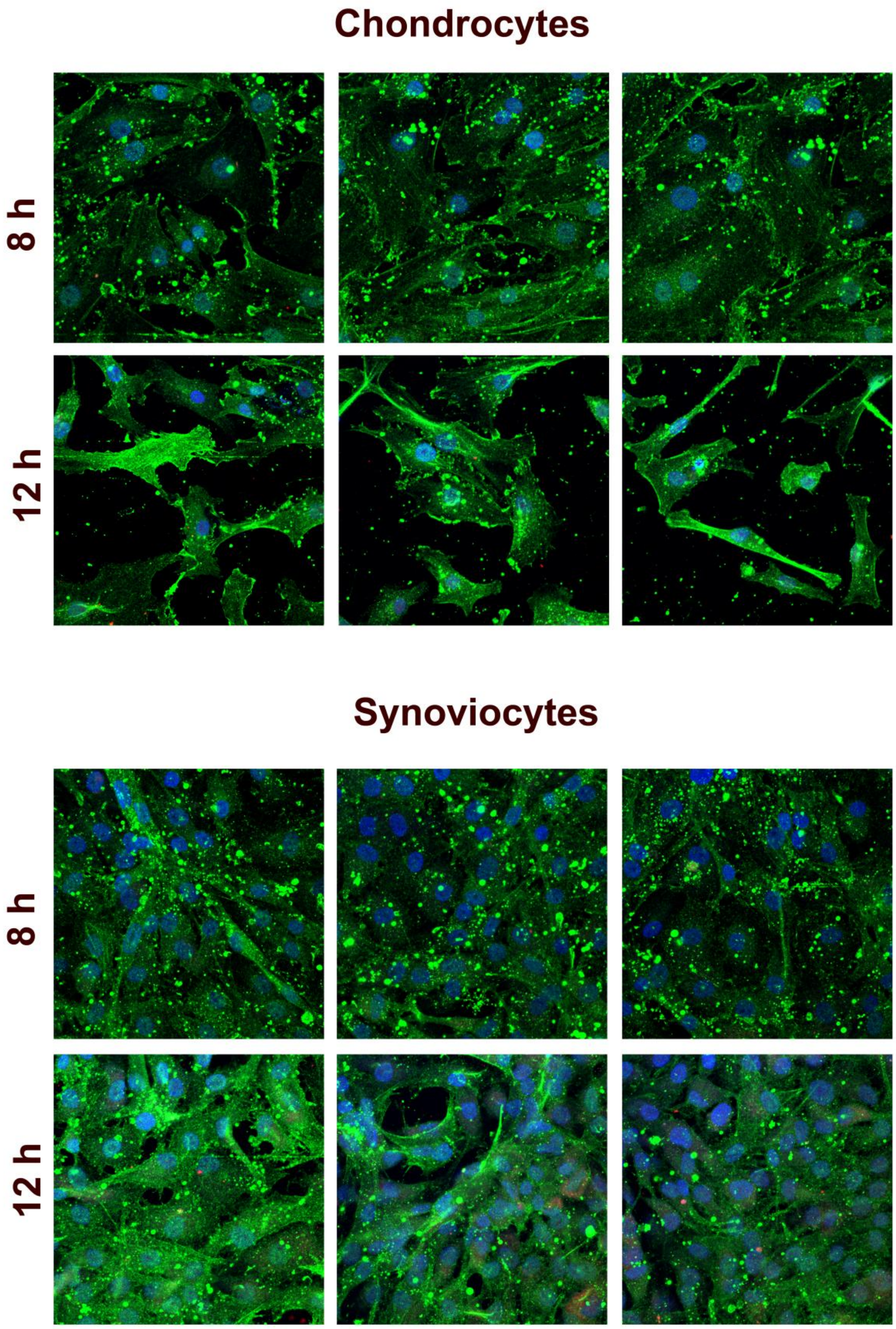

S3

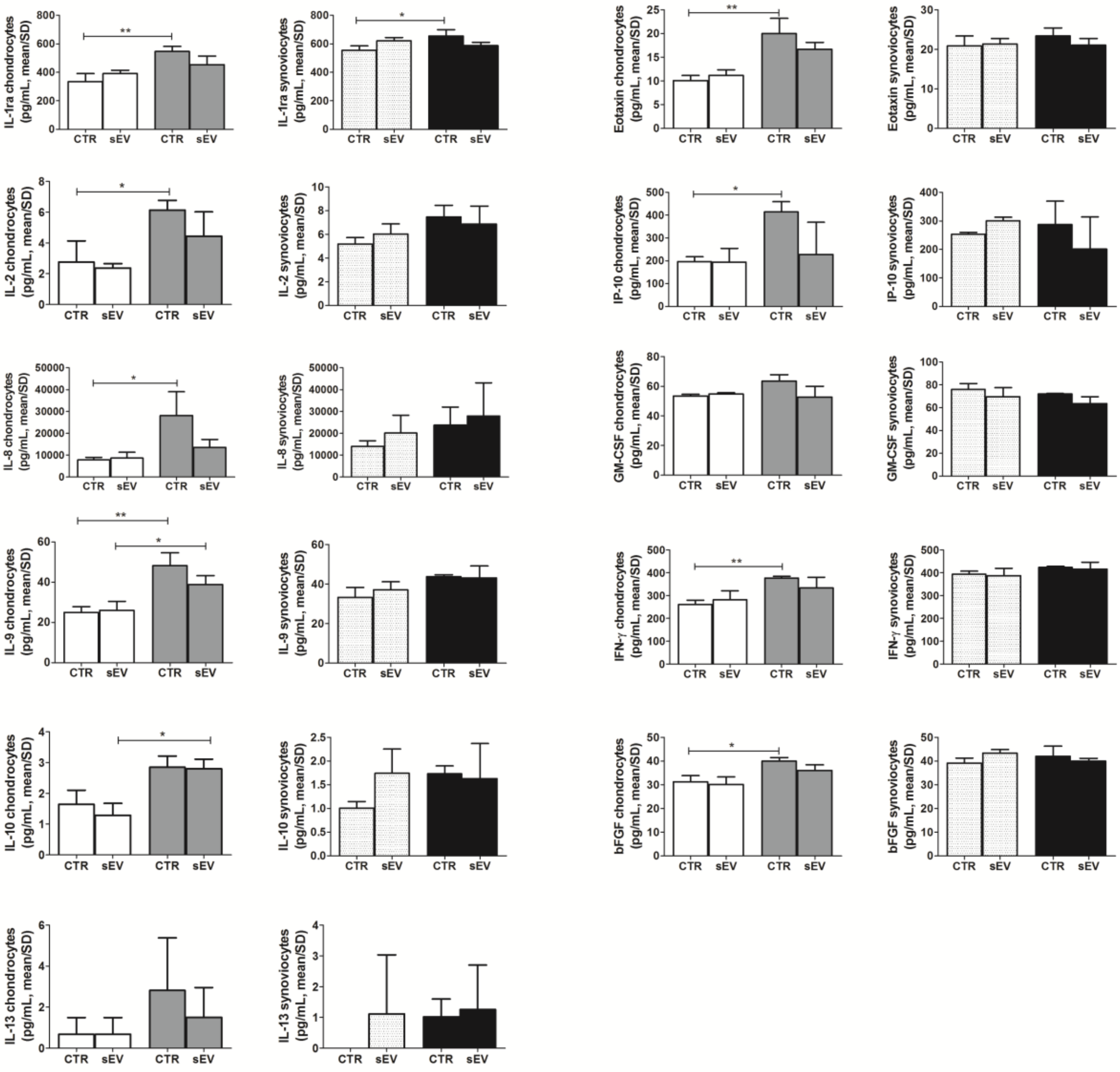
IL-1ra chondrocytes (pg/mL, mean/SD)
IL-1ra synoviocytes (pg/mL, mean/SD)
Eotaxin chondrocytes (pg/mL, mean/SD)
Eotaxin synoviocytes (pg/mL, mean/SD)
IL-2 chondrocytes (pg/mL, mean/SD)
IL-2 synoviocytes (pg/mL, mean/SD)
IP-10 chondrocytes (pg/mL, mean/SD)
IP-10 synoviocytes (pg/mL, mean/SD)
IL-8 chondrocytes (pg/mL, mean/SD)
IL-8 synoviocytes (pg/mL, mean/SD)
GM-CSF chondrocytes (pg/mL, mean/SD)
GM-CSF synoviocytes (pg/mL, mean/SD)
IL-9 chondrocytes (pg/mL, mean/SD)
IL-9 synoviocytes (pg/mL, mean/SD)
IFN-γ chondrocytes (pg/mL, mean/SD)
IFN-γ synoviocytes (pg/mL, mean/SD)
IL-10 chondrocytes (pg/mL, mean/SD)
IL-10 synoviocytes (pg/mL, mean/SD)
bFGF chondrocytes (pg/mL, mean/SD)
bFGF synoviocytes (pg/mL, mean/SD)
IL-13 chondrocytes (pg/mL, mean/SD)
IL-13 synoviocytes (pg/mL, mean/SD)
CTR sEV CTR sEV

S4

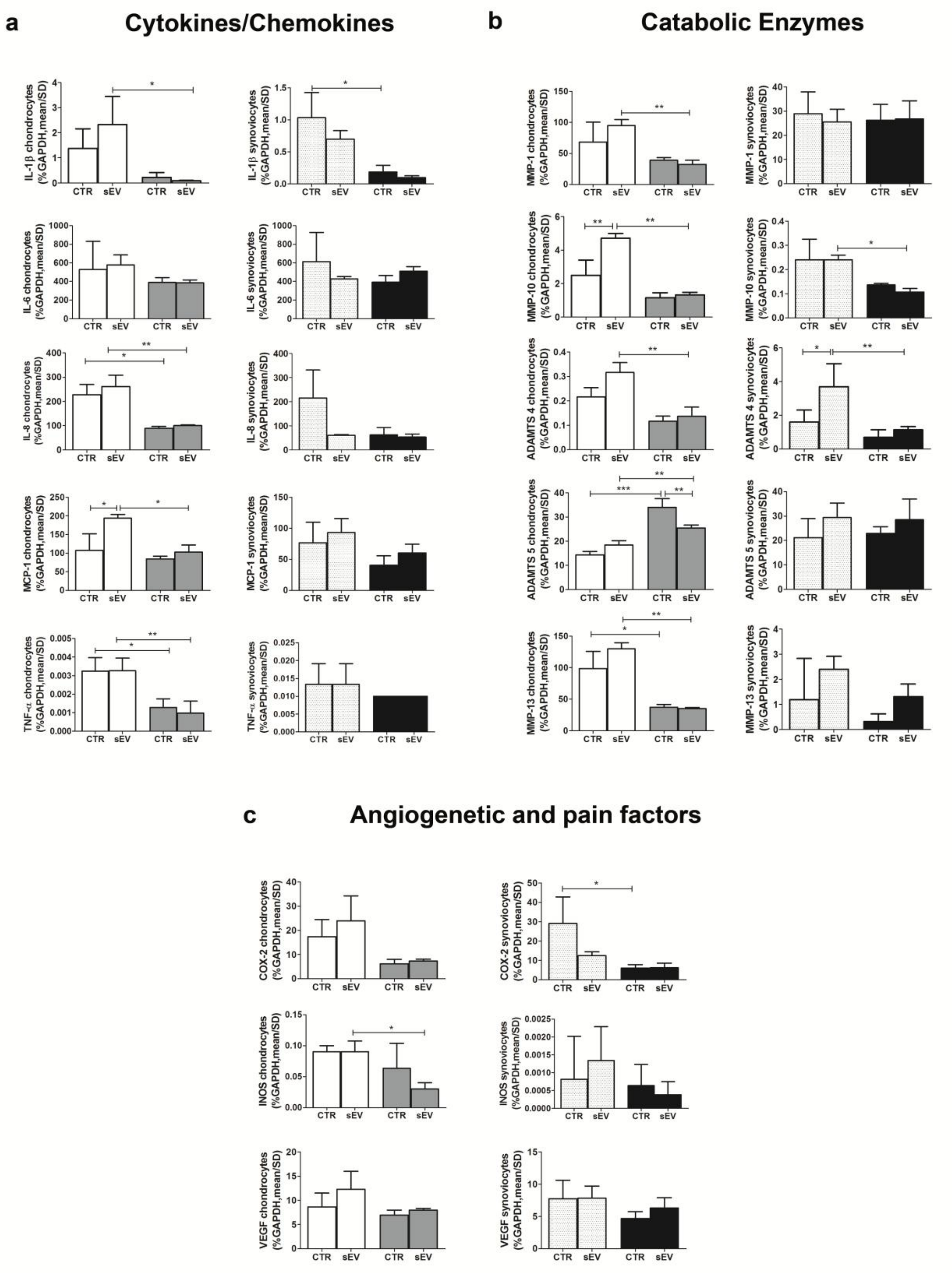
a
Cytokines/Chemokines
b
Catabolic Enzymes
c
Angiogenetic and pain factors
IL-1β chondrocytes (%GAPDH,mean/SD)
IL-1β synoviocytes (%GAPDH,mean/SD)
IL-6 chondrocytes (%GAPDH,mean/SD)
IL-6 synoviocytes (%GAPDH,mean/SD)
IL-8 chondrocytes (%GAPDH,mean/SD)
IL-8 synoviocytes (%GAPDH,mean/SD)
MCP-1 chondrocytes (%GAPDH,mean/SD)
MCP-1 synoviocytes (%GAPDH,mean/SD)
TNF-α chondrocytes (%GAPDH,mean/SD)
TNF-α synoviocytes (%GAPDH,mean/SD)
MMP-1 chondrocytes (%GAPDH,mean/SD)
MMP-1 synoviocytes (%GAPDH,mean/SD)
MMP-10 chondrocytes (%GAPDH,mean/SD)
MMP-10 synoviocytes (%GAPDH,mean/SD)
ADAMTS 4 chondrocytes (%GAPDH,mean/SD)
ADAMTS 4 synoviocytes (%GAPDH,mean/SD)
ADAMTS 5 chondrocytes (%GAPDH,mean/SD)
ADAMTS 5 synoviocytes (%GAPDH,mean/SD)
MMP-13 chondrocytes (%GAPDH,mean/SD)
MMP-13 synoviocytes (%GAPDH,mean/SD)
COX-2 chondrocytes (%GAPDH,mean/SD)
COX-2 synoviocytes (%GAPDH,mean/SD)
INOS chondrocytes (%GAPDH,mean/SD)
INOS synoviocytes (%GAPDH,mean/SD)
VEGF chondrocytes (%GAPDH,mean/SD)
VEGF synoviocytes (%GAPDH,mean/SD)
CTR
sEV

S5

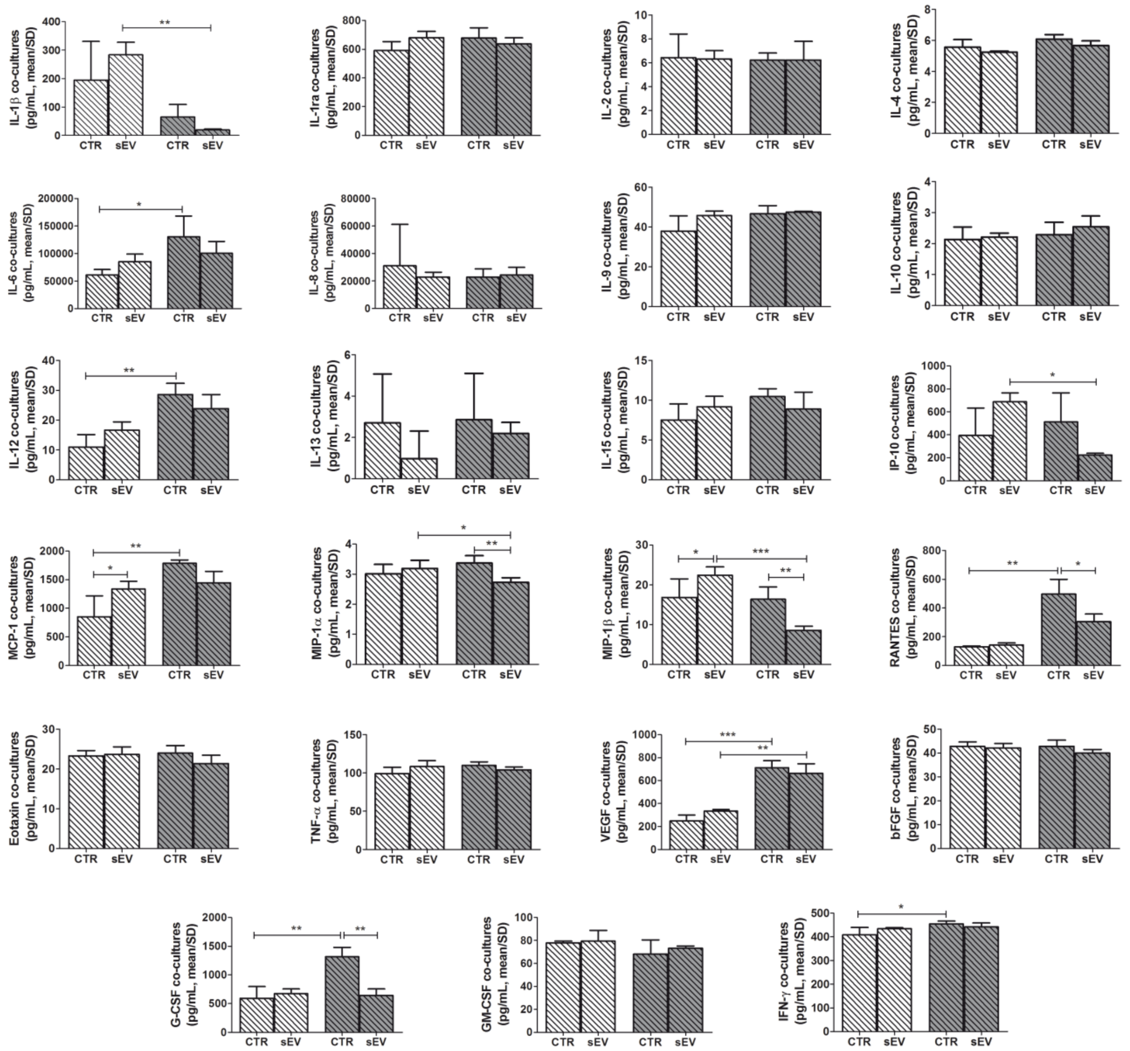

S6

A

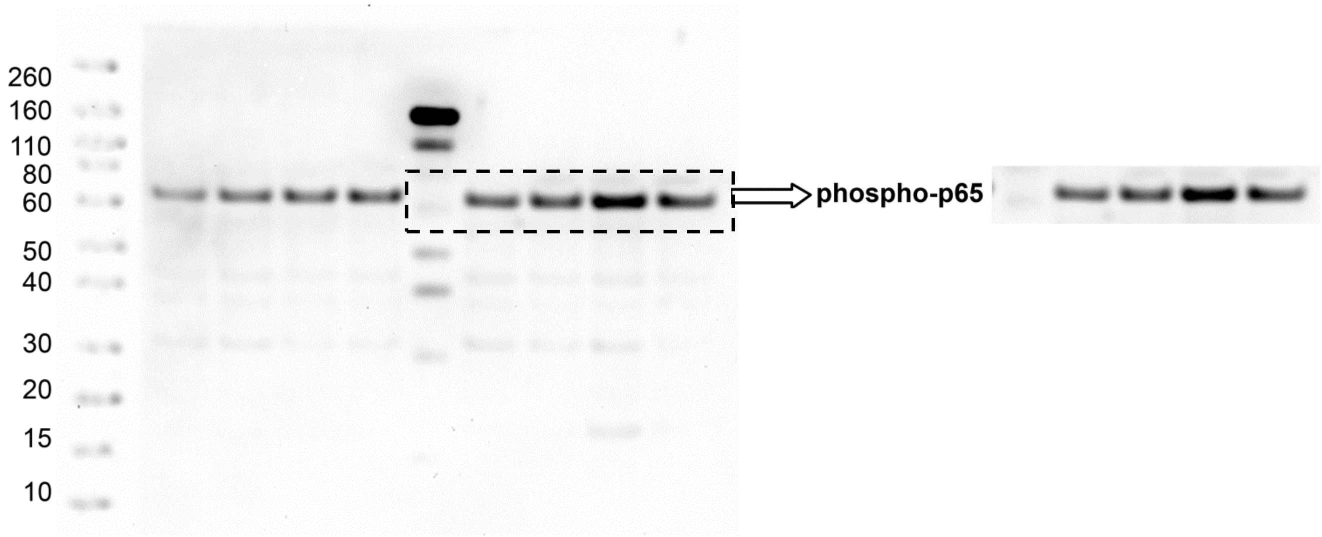
260
160
110
80
60
50
40
30
20
15
10
phospho-p65

B

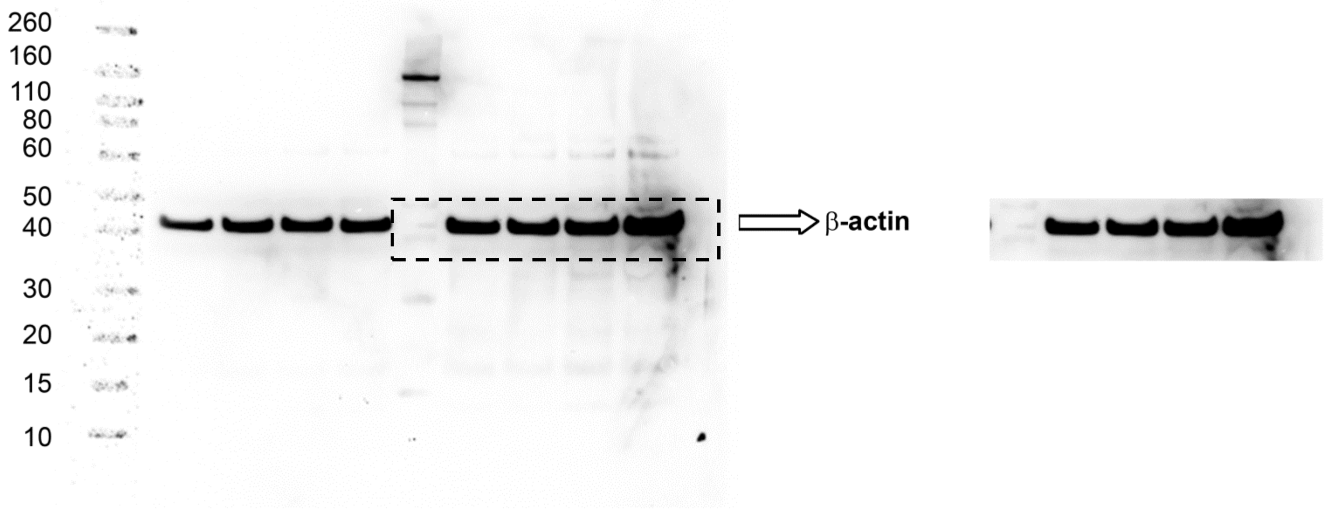
260
160
110
80
60
50
40
30
20
15
10
β-actin

S7

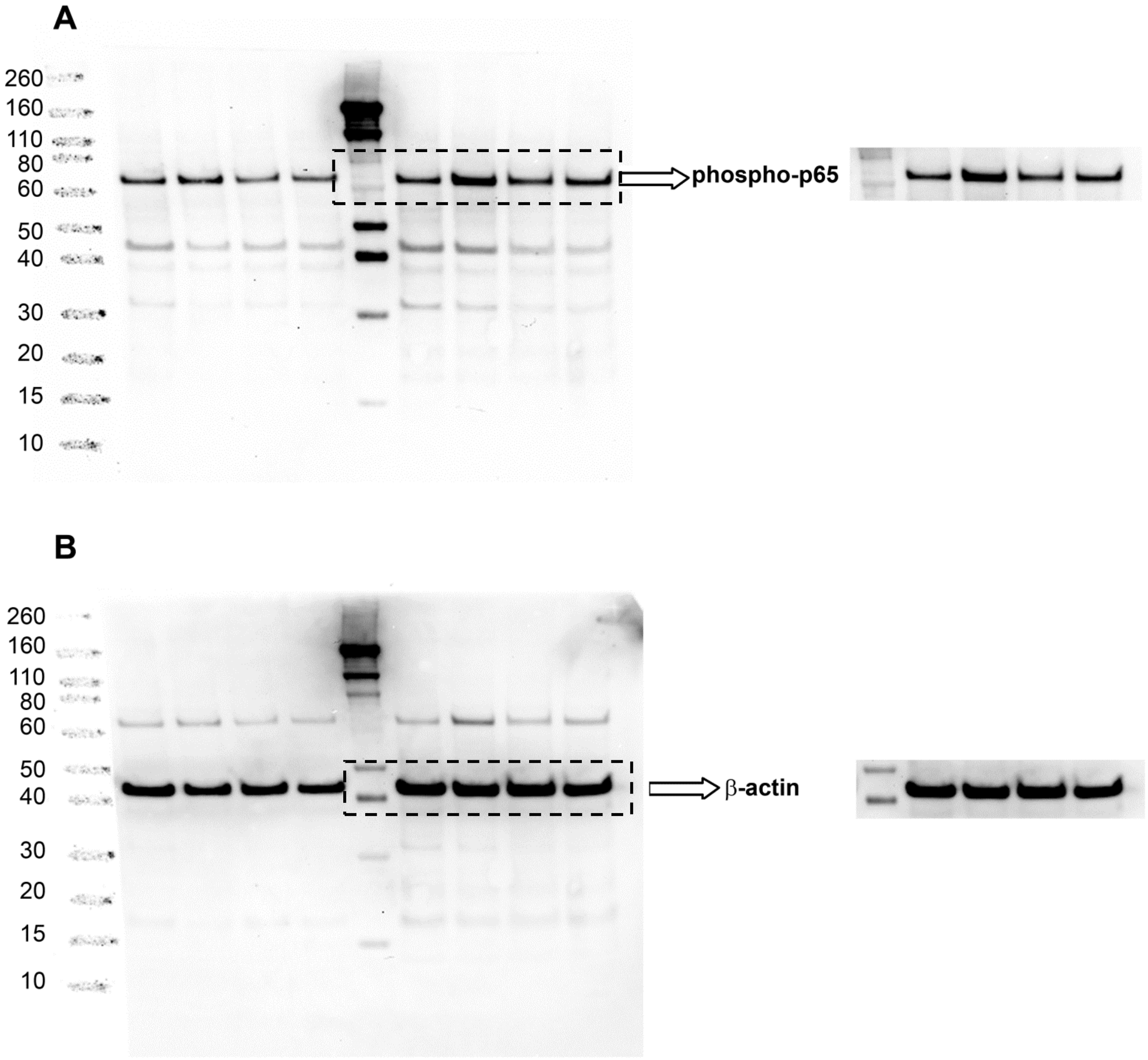
A
260
160
110
80
60
50
40
30
20
15
10
phospho-p65
B
260
160
110
80
60
50
40
30
20
15
10
β-actin

LEGEND TO SUPPLEMENTARY FIGURES

Figure S1: Full blots used to derive the calnexin and golgin results shown in Figure 1d of the main manuscript, obtained with lysates of adipose derived stromal cells and purified exosomes loaded in NuPAGE Novex 4-12% Bis-Tris gels. Samples were run with NuPAGE MOPS along with Novex Sharp Pre-Stained Protein Standards. After protein transfer, the lanes containing the standards were cut from the membranes containing the samples. To assess the molecular weight of western blot stained bands the pre-stained bands of the markers were highlighted by mean of a Glow Writer pen (http://divbio.com/glow-writerpen.aspx) and at the end of western blotting, the lanes containing the Protein Standards were juxtaposed to the original membranes. The dashed rectangles indicates the bands included in the crops, and on the right the arrow indicates the bands used to set up Figure 1d.

Figure S2. To confirm the specificity of the images shown in Figure 2, a "blank" control was carried out. Indeed, the long aliphatic dye PKH26 is prone to form micelles of similar size to small Extracellular Vesicles. Therefore, a labeling experiments was carried out using PKH26 at the same concentration used in experiments shown in Figure 2 (4 µM) and the labeled material was applied at both chondrocytes and synoviocytes and left for both 8 and 12 hours, time points that gave the strongest evidence of vesicle uptake in Figure 2. Then the samples were fixed and stained as described at section 2.9 of the manuscript, with visualization of the cells by means of actin staining and DAPI nuclear counterstaining. Three images were taken at each time points for both chondrocytes and synoviocytes, that indicate only sporadic occurrence of red spots at a negligible level compared to what shown in Figure 2.

Figure S3. EXO effects on Cytokines/Chemokines protein release from chondrocytes (left graphs: white histograms, CTR and EXO conditions at 4 hours, and grey histograms: CTR and EXO conditions at 15 hours) and synoviocytes (right graphs: lighter histograms, CTR and EXO conditions at 4 hours, and black histograms: CTR and EXO conditions at 15 hours). Data were expressed as mean ± SD. These results have been obtained by mean of the Bio-Plex Protein Array System and integrate those shown in Figure 4 of the manuscript. Statistical analysis was performed by mean of the GRAPHPAD Prism 5.0 software. Since comparisons were undertaken among multiple groups (CTR- IL-1β-treated and EXO treatment of IL-1 stimulated samples at 4 and 15 hours) the means of the groups were compared by mean of ANOVA, followed by Tukey's post hoc test. The differences were considered significant when $P < 0.05$ with *$P < 0.05$; **$P < 0.01$; and ***$P < 0.001$.

Figure S4. EXO effects on gene expression of Cytokines/Chemokines (A), Catabolic Enzymes (B) and Angiogenetic and Pain Factors (C) in both chondrocytes and synoviocytes grown in co-culture. Data were normalized to GAPDH and expressed as mean ± SD. Left graphs are related to chondrocytes while right graphs are related to synoviocytes. Different pattern are used for different cells and time points: chondrocytes at 4h, white; chondrocytes at 15 h, gray; synoviocytes at 4h, fine dotted pattern; synoviocytes at 15 h, black. The means of the groups were compared by mean of ANOVA, followed by Tukey's post hoc test. The differences were considered significant when $P < 0.05$ with $*P < 0.05$; $**P < 0.01$; and $***P < 0.001$.

Figure S5. EXO effects on Cytokines/Chemokine protein release from cocultures. Data were expressed as mean ± SD. The dashed pattern indicates cocultures. Different filling colours are used for different time points: 4h: white; 15 h: gray. The means of the groups were compared by mean of ANOVA, followed by Tukey's post hoc test. The differences were considered significant when $P < 0.05$ with $*P < 0.05$; $**P < 0.01$; and $***P < 0.001$.

Figure S6 and figure S7: Full blots used to derive the original phospho-p65 results shown in Figure 5c of the main manuscript (Figure S6A and Figure S7A), along with β-actin, as loading control (Figure S6B and Figure S7B). These results were obtained with lysates from chondrocytes (Figure S6) and synoviocytes (Figure S7) in CTR (IL-1β treated) or sEV (IL-1β+sEV treated) conditions at 4 and 15 h. The dashed rectangles indicate the bands included in figure 5 and on the right the arrows indicate the insets used to set up Figure 5c.
